# Post-Functionalization of Organometallic Complexes via Click-Reaction

**DOI:** 10.3390/molecules27196494

**Published:** 2022-10-01

**Authors:** Stanislav Petrovskii, Viktoria Khistiaeva, Aleksandra Paderina, Evgenia Abramova, Elena Grachova

**Affiliations:** Institute of Chemistry, St. Petersburg University, 198504 St. Petersburg, Russia

**Keywords:** click-reaction, CuAAC, platinum complex, iridium complex, rhenium complex, post-synthetic modification

## Abstract

CuAAC (Cu catalyzed azide-alkyne cycloaddition) click-reaction is a simple and powerful method for the post-synthetic modification of organometallic complexes of transition metals. This approach allows the selective introduction of additional donor sites or functional groups to the periphery of the ligand environment. This is especially important if a metalloligand with free donor sites, which are of the same nature as the primary site for the coordination of the primary metal, has to be created. The concept of post-synthetic modification of organometallic complexes by click-reaction is relatively recent and the currently available experimental material does not yet allow us to identify trends and formulate recommendations to address specific problems. In the present study, we have applied the CuAAC reaction for the post-synthetic modification of diimine mononuclear complexes Re(I), Pt(II) and Ir(III) with C≡C bonds at the periphery of the ligand environment and demonstrated that click-chemistry is a powerful tool for the tunable chemical post-synthetic modification of coordination compounds.

## 1. Introduction

Transition metal complexes are interesting objects from both fundamental and application points of view and their practically useful properties are determined by the combination of the metal center and ligand environment. To date, there are a large number of applications requiring the incorporation of a transition metal coordination compound into a complex molecular system by covalent conjugation without loss of practically useful properties. This problem can potentially be addressed by two synthetic pathways: (a) utilization of a ready-to-use donor site for metal coordination and (b) introducing a functional group on the periphery of the ligand environment for conjugation to the complementary substrate. The latter approach is a post-synthetic modification/functionalization of the complex and can be realized with high selectivity.

**Scheme 1:**
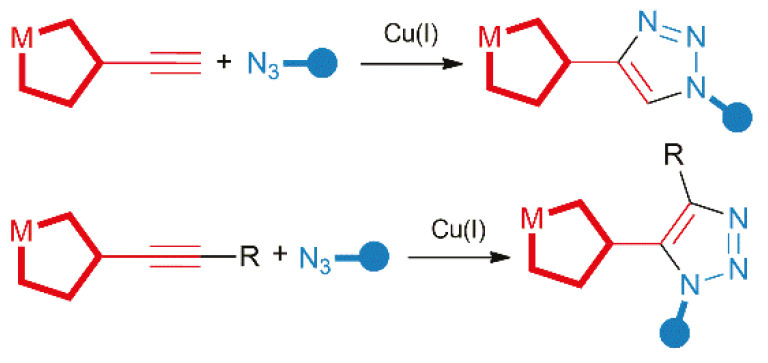
Schematic representation of CuAAC reaction between functionalized metal complex and substituted organic azide.

Triazoles are convenient scaffolds [1] that can have various substituents independent of nature (organic, inorganic, polymeric, nanoscale, biological, etc.). Two different fragments can be combined to form a triazole ring by the CuAAC (Cu catalyzed azide-alkyne cycloaddition) click-reaction [2,3], the best known and the most widely used form of the click processes. Despite the generally accepted success and undoubted potential in the construction of complex molecular ensembles, this approach is relatively rarely used in the field of coordination chemistry. However, the philosophy and methodology of click-chemistry are entering deeper into daily synthetic coordination chemistry, in particular, it is a convenient synthetic approach for creating new ligands [4,5]. A natural expansion of the idea of click-chemistry in the field of coordination chemistry is the creation of complex molecular objects by introducing click-groups (in particular, terminal C≡C bond) at the periphery of the ligand sphere for further click-conjugation (Scheme 1).

To date, a number of complexes containing C≡C bond on periphery of ligand environment to be involved in click-reaction have been reported in the literature for Mo(I), Tc(I, V) and Re(I, V) [6,7,8,9,10,11], Ir(III) [12,13,14,15], Pt(II) [16,17,18,19,20,21,22,23], Pt(IV) [24], Ru(II, III) [25,26,27,28,29,30,31,32,33,34], Fe(II) [35,36], ferrocene derivatives [37,38,39,40,41], Mn(I) [42], Ni(II) [43], Zn(II) porphyrins and analogues [44,45,46,47], Cr(0) and W(0) [48], Au(I) [49], Ln(III) [50,51,52,53,54,55,56], borane derivatives [57,58], polyoxometalates (POM) [59,60]. The click-chemistry approach allows the creation of multifunctional luminescent complexes [61], construction of conjugates for precision bio-imaging and diagnostics [62], attachment of vectors for transport in biological systems [63], creation of DNA-based materials, attachment of long hydrocarbon chains, attachment of electronic reservoirs for photocatalysis, construction of homo- and heterometallic bridging complexes, functionalization of polymers [64,65], and modification of MOFs [66] (representative examples are summarized in Table A1. 

However, any evaluations of the metallocentre nature influence on the efficiency of the click-reaction have not received sufficient attention, although there is mention that post-modification can depend on the nature of the metal [67]. Thereby, the understanding of fundamental aspects of the click-chemistry approach for post-functionalization of transition metal complexes is still an open problem, and it is critically important for the rational molecular design of smart functional materials. 

In the present work, we report a proof-of-principle solution for click-conjugation via CuAAC reaction of 2-azidomethylpyridine with the most popular luminescent diimine mononuclear complexes of Re(I), Pt(II) and Ir(III) bearing terminal C≡C bond on a periphery of the ligand environment and provide support that the click-chemistry is a powerful tool for the chemical post-synthetic modification of coordination compounds. The work presented herein is a contribution to the data bank to support the development of experimental approaches to the post-functional modification of transition metal complexes.

## 2. Results

### 2.1. Synthesis of Functionalized NN Compounds 1^P^−3^P^

**Scheme 2:**
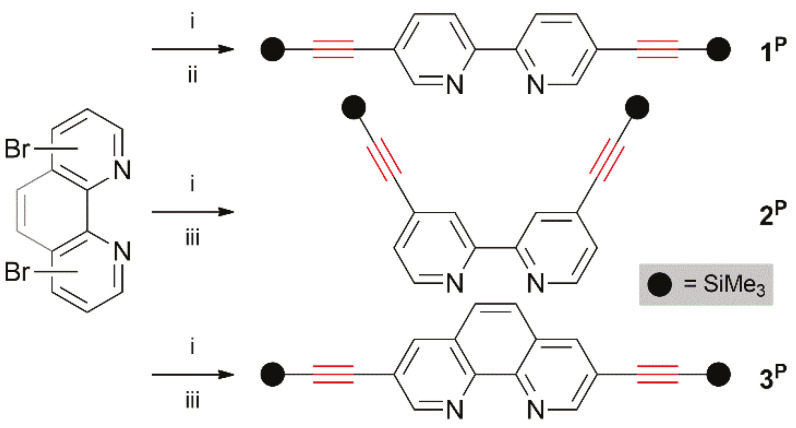
Synthesis of functionalized **NN** compounds **X^P^** (X = 1–3). i = Pd(PPh_3_)_2_Cl_2_, CuI, HN(^i^Pr)_2_, (CH_3_)_3_SiC_2_H; ii = THF, RT, 24 h; iii = THF, 45 °C, 72 h.

The substituted bipyridines **1^P^** and **2^P^** were synthesized based on the slightly modified literature methods (Scheme 2) [68]. The other method based on previously published work was developed for the synthesis of compound **3^P^** [69]. It should be noted that the yield of compound **3^P^** is low and the ratio “cost of the synthesis/production of the target compound in pure form” is not justified for this potentially interesting compound. The main problem using the literature methods for the synthesis of **NN** compounds with C≡CH functional groups was that the products were contaminated with phosphine oxide and traces of Cu(II). Both of these impurities could affect the further production of the complexes, so a purification technique was specifically developed to remove these impurities completely. Since unprotected terminal acetylene is able to polymerize in the presence of transition metals, compounds **X^P^** (X = 1–3) with TMS protected acetylene units were directly used for the synthesis of transition metal complexes.

### 2.2. Synthesis and Post-Functionalization of Re(I) Complexes

**Scheme 3:**
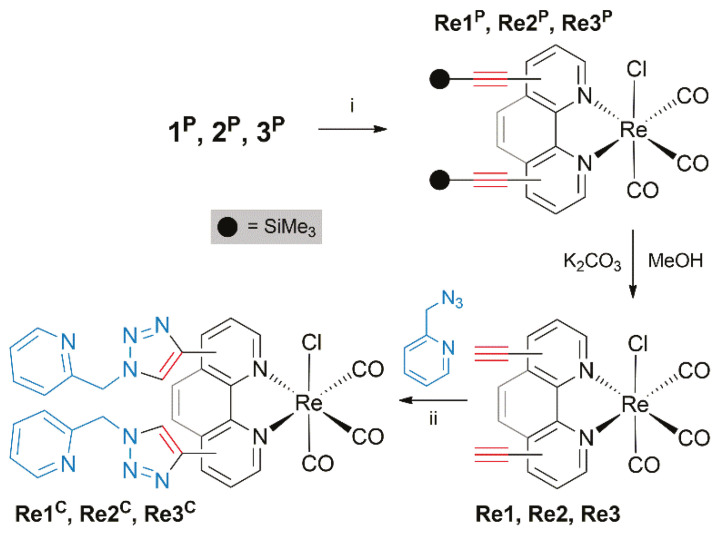
Synthesis and post-functionalization of Re(I) complexes. i = Re(CO)_5_Cl, refluxing toluene, 3 h; ii = CuSO_4_×5H_2_O, NaAsc, acetone/H_2_O, r. t., 12 h.

The archetypal Re(I) heteroleptic neutral complexes **ReX^P^** (X = 1−3) decorated by protected **NN** ligand were obtained in the canonical reaction between Re(CO)_5_Cl and **X^P^** compound in refluxing toluene and in good yields (Scheme 3). Deprotection of the C≡C bond occurs in very mild conditions in methanol solution in the presence of K_2_CO_3_ at room temperature and takes 4 h to be completed resulting in complexes **ReX** (X = 1−3) formation. The following CuAAC reaction of post-functionalization of **ReX** complexes by 2-azidomethylpyridine also does not request any hard conditions and carries out in an acetone/water mixture in the presence of CuSO_4_ and NaAsc (sodium ascorbate) at room temperature overnight, resulting in **ReX^C^** (X = 1−3) formation with good yield. Complexes of **ReX^C^** are microcrystalline yellow solids that are moisture and air-stable, and readily soluble in common polar solvents such as chloroform, dichloromethane, acetone, and methanol. 

The composition and structures of **ReX^P^**, **ReX** and **ReX^C^** (X = 1−3) were established by proton NMR experiments and ESI MS (in positive mode). On the other side, ^1^H NMR spectroscopy allowed us to control the transformation of the Re(I) complexes. The number of the signals in ^1^H NMR spectra, their relative integral intensities and multiplicities clearly indicate the presence of chelating and symmetrically substituted **NN** ligand in the coordination environment of Re(I) complexes (Figures S2–S4). 

The complete disappearance of resonance that was assigned to protons of protective SiMe_3_ groups in **ReX^P^** spectra ca. 0.3 ppm and appearance of signal responsible for acetylene protons ca. 4 ppm in proton NMR spectra provide support for complete deprotection of C≡C bond in coordination environment of **ReX^P^** complexes and formation of **ReX** complexes. The successful click-reaction and **ReX^C^** complexes formation are verified by a set of resonances of triazole and pyridine rings in the aromatic part of proton NMR spectra. 

ESI^+^ MS spectra of the Re(I) complexes display the signals of [M – Cl]^+^, [M + Na]^+^ and [M – Cl + N_2_]^+^ molecular ions with expected m/z values and isotopic patterns matching the calculated ones (Figures S5–S7).

### 2.3. Synthesis and Post-Functionalization of Ir(III) Complexes

**Scheme 4:**
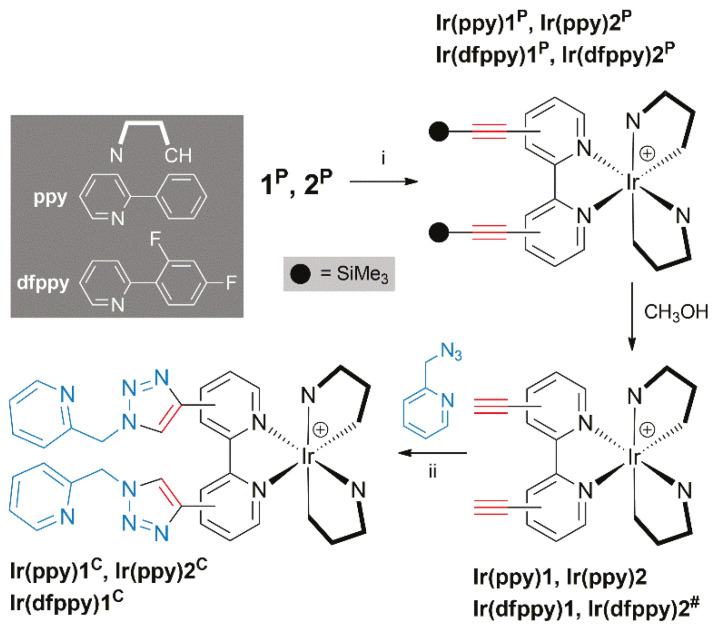
Synthesis and post-functionalization of Ir(III) complexes. i = [Ir(C^^^N)_2_Cl]_2_, CH_2_Cl_2_/MeOH, excess of KPF_6_, r.t., 24 h; ii = CuSO_4_×5H_2_O, NaAsc, acetone/H_2_O, r.t., 48 h. ^#^ Reaction uncompleted.

The archetypal Ir(III) heteroleptic cationic complexes decorated by two cyclometallating ligands and ‘clickable’ diimine ligands were obtained in the canonical reaction between dichloro-bridged dimer complexes and **X^P^** compounds under mild conditions (Scheme 4). The cyclometallating ligands **ppy** and **dfppy** were chosen because of the difference in their donor-acceptor properties. The composition and structures of the Ir(III) complexes under study were established by proton NMR experiments (Figures S8–S11) and ESI^+^ MS (Figures S12–S15). Likewise, for all complexes studied herein, spectroscopy data provide support that the composition and structure of Ir(III) complexes are as expected. 

It is important to note that, unlike all other complexes, the deprotection of Ir(III) complexes occurs by simple dissolution in methanol. The exception is **Ir(dfppy)2^P^**, for which deprotection unexpectedly does not complete under these conditions even for a long reaction time and in the presence of KF. Moreover, the addition of K_2_CO_3_ or KOH leads to the destruction of the complex. ^1^H NMR data for complex **Ir(dfppy)2** were obtained by subtracting the spectrum of complex **Ir(dfppy)2^P^** from the spectrum of the reaction mixture (Figure S11). 

The click-reaction of **Ir(ppy)1**, **Ir(ppy)2** and **Ir(dfppy)1** with 2-azidomethylpyridine takes a longer time than the one for Re(I) complexes described above and results in the formation of complexes **Ir(ppy)1^C^**, **Ir(ppy)2^C^** and **Ir(dfppy)1^C^** with relatively lower yields. There is a dependence of the reaction yield on the nature of the cyclometallating ligand, and this value is higher for the more acceptor **C^^^N** ligand (72% for **Ir(dfppy)1^C^** vs. 45% and 48% for **Ir(ppy)1^C^** and **Ir(ppy)2^C^**, respectively). 

### 2.4. Synthesis and Post-functionalization of Pt(II) Complexes

**Scheme 5:**
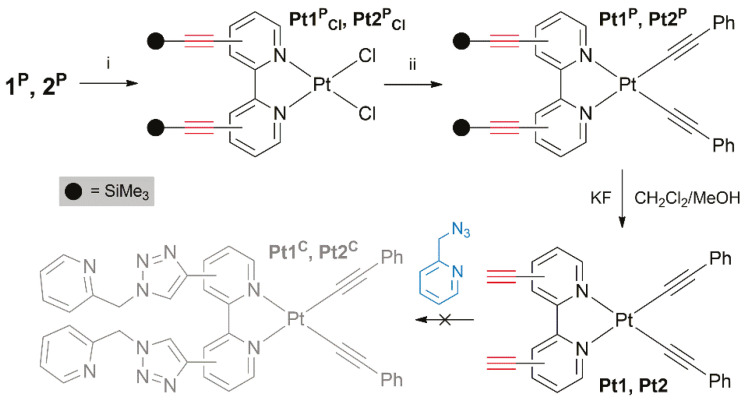
Synthesis of Pt(II) complexes. i = (DMSO)_2_PtCl_2_, MeCN, r.t.; ii = C_2_Ph, CuI, ^i^Pr_2_NH, CH_2_Cl_2_, r.t., 12 h.

Pt(II) compounds **PtX^P^** (X = 1, 2) were prepared from dichloride starting compounds according to the published protocol [19,70] through attaching of alkynyl ligands by Sonogashira coupling reaction that is the general method used for similar Pt(II) derivatives (Scheme 5). As for the Re(I) complexes described above, the deprotection of the C≡C bond occurs under very mild conditions in methanol solution at room temperature but using KF instead of carbonate gives higher **PtX** (X = 1, 2) yields. However, in contrast to our expectations and published data [19,21], the click-reactions of **PtX^P^** or **PtX** complexes with 2-azidomethylpyridine did not result in the formation of **PtX^C^**. We tested various conditions (solvent, time and Cu(I) catalyst), but only an unidentified mixture of compounds was obtained. 

**Scheme 6:**
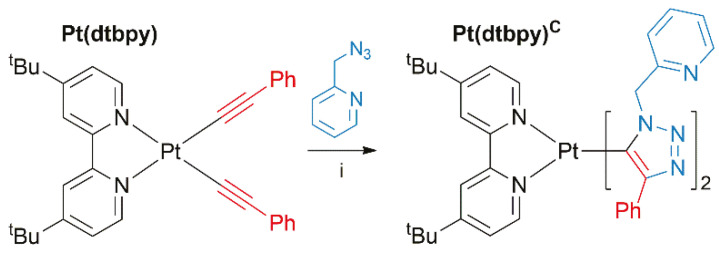
Post-functionalization of **Pt(dtbpy)** complex. i = CuSO_4_ × 5H_2_O, NaAsc, CH_2_Cl_2_/MeOH, 40 °C, 48 h.

Supposing that the σ-alkynyl ligands can be non-innocent, we replaced the C≡C substituents in the **NN** ligand with a ^t^Bu group and performed a test click-reaction with 2-azidomethylpyridine. In this case, the reaction takes a slight heating and 48 h to be completed and resulting in the formation of only one product, namely **Pt(dtbpy)^C^** complex (Scheme 6). 

The structures of **Pt(dtbpy)^C^** were established by ^1^H NMR experiment, ESI MS (positive mode), and single crystal X-ray diffraction (XRD) analyses. 

The number of signals in ^1^H NMR spectrum, their relative integral intensities and multiplicities (Figures S18 and S19) clearly indicate the presence of **NN** ligand and two triazol rings substituted of methylpyridine and phenyl that confirm that both C≡C bonds have reacted with 2-azidomethylpyridine in contrast to the early reported unsymmetrical product obtained in a similar process [20]. ESI^+^ MS spectra of **Pt(dtbpy)^C^** complex demonstrate the signals of [M + H]^+^ and [M + Na]^+^ molecular ions with expected m/z values and isotopic patterns matching the calculated ones (Figure S20).

**Figure 1:**
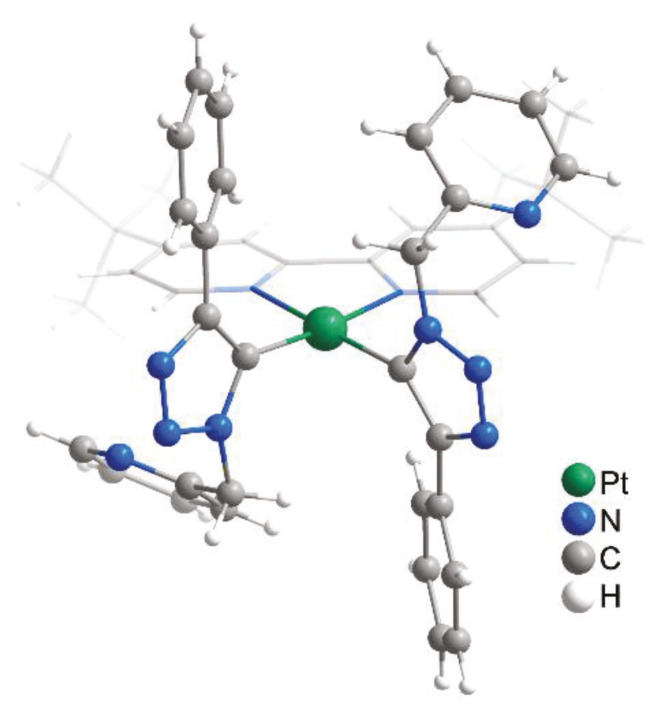
Molecular structure of **Pt(dtbpy)^C^** complex.

The molecular structure of **Pt(dtbpy)^C^** is shown in Figure 1 and Figure S21; crystallographic data are given in Table S1, and selected structural parameters are listed in Table S2. The molecular arrangement of Pt(II) center found in the crystals correlates well with spectroscopic data obtained for **Pt(dtbpy)^C^** in solution. According to the XRD data, the platinum metallocentre possesses a square-planar environment, with a chelating ring formed after the coordination of **dtbpy** ligand, and two monodentate triazolyl rings. To the best of our knowledge, there is only one example of platinum triazolyl-bipyridine complexes, already mentioned [20], but it is unsymmetrical. There are also three more examples of triazolyl Pt(II) complexes, bearing ancillary tridentate ligands. [71] Being the very first example of bis-triazolyl Pt(II) complexes, **Pt(dtbpy)^C^**, however, can be compared to the above-mentioned four compounds, as they possess close structural units. Selected structural parameters for the complex described are listed in Table S2. 

The bond lengths Pt1–N1 and Pt1–N2 in **Pt(dtbpy)^C^** fall into the literature range. On the contrary, the bond Pt1–C19 is slightly longer than described in the literature (Table S2). Bond lengths of triazolyl rings also fall in the literature range. The plane of the triazolyl ring is almost perpendicular to the Pt-dtbpy plane. Pyridine rings are placed in opposite directions, presumably due to steric hindrances. Complex **Pt(dtbpy)^C^** is packed in a layered fashion with no π-π interactions. 

### 2.5. Photophysical Properties of Functionalized Complexes

Diimine complexes of Re(I),Pt(II) and Ir(III) are luminescent, and the electronic states of the **NN** ligand are usually involved in the transitions responsible for photoemission. In order to understand how the post-synthetic modification of the **NN** ligand affects the luminescence properties of the complex, we investigated their basic photophysical characteristics (Table S3). 

**Figure 2:**
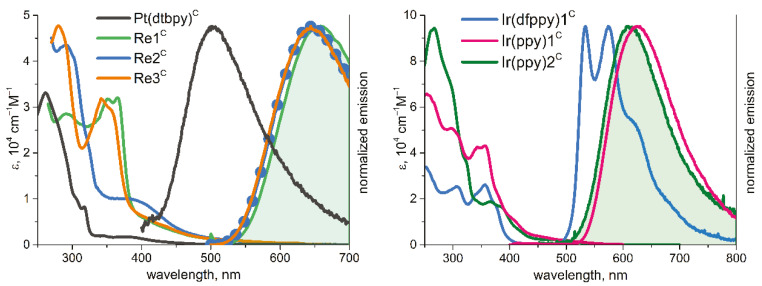
Absorption spectra and normalized emission (λ_ext_ 365 nm) spectra of Re(I), Pt(II), and Ir(III) “clicked” complexes, DCE solution, 10^−5^ M, r.t.

The UV-vis absorption spectra of “clicked” complexes (Figure 2) are typical for carbonyl Re(I), alkynyl Pt(II) and cyclometallated Ir(III) complexes with **NN** ligands, and exhibit strong bands in UV range from ca. 250 to ca. 400 nm due to spin-allowed ππ* intraligand IL transitions located at the aromatic system of ligands. The low energy edge from ca. 400 to ca. 500 nm range may include the contribution of ligand-to-ligand charge transfer LLCT as well as the MLCT transitions from metal dπ-orbitals to π*-orbital of ligands. 

All complexes studied exhibit emission in the visible region of the spectrum when excited by UV light. The large Stokes shift, oxygen sensitivity and afterglow lifetime in the microsecond domain clearly indicate a triplet origin of the luminescence, which is typical for this kind of compound. The post-synthetic modification of the **NN** ligand has a minor effect on the emission energy for Ir(III) complexes, which undergoes a non-significant bathochromic shift ca. 20 nm compared to [Ir(ppy)_2_(bpy)]^+^ (λ_em_ ca. 600 nm) [72,73] and [Ir(dfppy)_2_(bpy)]^+^ (λ_em_ ca. 520 nm) [74]. At the same time, the emission energy of the Re(I) complexes undergoes a significant bathochromic shift up to 80 nm compared to Re(CO)_3_X(bpy) and Re(CO)_3_X(phen), X = Cl, Br [75].

The post-synthetic conversion of **Pt(dtbpy)** in **Pt(dtbpy)^C^** involves not **NN** ligand but σ-coordinated C≡C bond. In contrast to the complexes described above, the emission energy of **Pt(dtbpy)^C^** undergoes a hypsochromic shift compared to the luminescence energy of **Pt(dtbpy)** [76] and the closest analog Pt(II) complex with one clicked C≡C bond [20].

## 3. Discussion

The application of the “*chelate, then click*” strategy [8] allows post-synthetic modification of the ligand sphere of transition metal complexes with functional groups reactive to azide. In our study, 2-azidomethylpyridine was used to demonstrate a proof-of-principle solution for the selective creation of an additional donor site on the periphery of a complex, and the complementary part for the triazole ring assembly was a terminal C≡C bond attached to the aromatic system of **NN** ligand (Scheme 1). Thus, the efficiency of the synthetic approach for different metallocentres has been illustrated. We do not pretend to a comprehensive conclusion and consider the results of our study as a contribution to the data bank to support the development of experimental approaches to the post-functional modification of transition metal complexes. 

For all reaction systems studied here, a mixture of copper sulfate pentahydrate and sodium ascorbate was used as the Cu(I) source to catalyze the click-reaction. This choice is traditional, however other Cu(I,II) salts or complexes can also be used (see Table A1). 

Based on the data obtained herein, Re(I) carbonyl complexes (Scheme 3) are the most convenient platform for post-functionalization by click-reaction. All three steps (preparation of the complex with protected C≡C bond on the periphery, deprotection, and click-reaction) are simple and occur under mild conditions with high yields. It is interesting to note that Re(I) complexes are also popular in the literature as convenient platforms for post-functionalization by click-reaction [6,10,11]. An important factor is that the information on the chemical behavior of rhenium complexes can be applied to similar technetium complexes, which is of fundamental importance for obtaining agents for radio-diagnostics [7,8,9]. 

Cyclometallated heteroleptic Ir(III) complexes with the composition [Ir(**C^^^N**)(**NN**)]^+^ represent one of the most popular molecular platforms for many applications due to their chemical stability and variability of luminescent properties. These complexes are ideal candidates for post-synthetic modification, including modification by click-reactions. However, the range of Ir(III) complexes with a terminal C≡C bond at the periphery of the ligand environment that are used for CuCAAC reaction is relatively limited [12,13,14,15]. The literature mainly describes the use of click-reaction to obtain a variety of triazole-based ligands for Ir(III) complexes, the range of which is huge. 

In the present study, two cyclometallating **C^^^N** ligands with different donor-acceptor properties were selected and four Ir(III) complexes with protected C≡C bonds at the periphery of the ligand environment were synthesized (Scheme 4). It has been found that the combination of two factors significantly influences deprotection as well as following click-reaction. These factors are the nature of the **C^^^N** ligand and the position of the C≡C bond in the aromatic ring of the **NN** ligand. In particular, deprotection of complex **Ir(dfppy)2^P^** (acceptor **C^^^N** ligand and C≡C bond in the *para*-position to the nitrogen atom) does not proceed smoothly and the click-reaction is very ineffective. Perhaps, the location of the triple bond in the *para*-position to the nitrogen atom in combination with the strong acceptor properties of the {Ir(**dfppy**)_2_} metallocentre leads to sufficient lowering of electron density on the triple bond, which results in its deactivation toward to the CuAAC reaction. 

In contrast to Re(I) (Scheme 3) and Ir(III) (Scheme 4) complexes, diimine Pt(II) complexes with σ-alkynyl ligands (Scheme 5) are objects of dual functionality. The presence of a terminal and σ-coordinated C≡C group increases the variability of the system since both types of triple carbon–carbon bonds can be click-reactive [20]. The literature examples demonstrate that the terminal C≡C group attached to the **NN** ligand has an advantage in the CuAAC reaction over the σ-alkynyl ligand [19,21,22] or the −C≡C− bond within the oligomer chain [16,17,18]. Our results are in contrast to this picture because no individual product was obtained in the click-reaction of **PtX** complexes with 2-azidomethylpyridine (Scheme 5). At the same time, click-reaction of the complex **Pt(dtbpy)** with the same substrate results in only one product (Scheme 6) with an acceptable yield. It is important to note that despite the external simplicity of the CuAAC reaction, this process is sensitive to many parameters including the nature of the azido-derivative. In particular, in the literature examples reviewed, this participant of the reaction does not have an additional donor site with high nucleophilicity (i.e., pyridine). 

## 4. Materials and Methods

### 4.1. General Comments

The ^1^H and ^1^H^1^H COSY NMR spectra were recorded on a Bruker 400 MHz Avance spectrometer (Daltonics, Bremen, Germany); chemical shifts were referenced to the residual solvent signals. Mass spectra were measured on a Bruker Daltonik MaXis HRMS-ESI-QTOF instrument (Daltonics, Bremen, Germany) operating in positive mode. Preparation of protected **NN** compounds **X^P^** (X = 1–3) [68,69] and **Re1^P^** [77] was carried on using the adapted protocol described in the literature, compounds **PtX^P^** and **PtX** (X = 1, 2) were prepared according to the published protocol [19,70]. [Pt(dtbpy)(C≡CPh)_2_] [78], 2-azidomethylpyridine [39], and cyclometalated Ir(III) dimers [Ir(C^N)_2_Cl]_2_ [79] were synthetized according to the published procedure. All other reagents and solvents were purchased from Merck (St. Louis, MO, USA), Alfa Aesar (Ward Hill, MA, USA), Fluka (Germany) and Vekton (St. Petersburg, Russia)) and used without further purification. 

### 4.2. Synthetic Methods

**5,5’-trimethylsilylethynyl-2,2’-bipyridine, 1^P^.** Schlenk flask was charged with 75 mg Pd(PPh_3_)_2_Cl_2_, 37,5 mg CuI, 375mg 4,4’-dibromo-2,2’-bipyridine, 25 mL of absolute THF, 580 µL trimethylsilylacetylene and 4 mL of diisopropylamine, then evacuated and filled with argon. The mixture was stirred at 45°C for 40 h. After the process was finished, the solvent was evaporated. Then the residue was dissolved in 25 mL of dichloromethane, and a small amount of activated carbon and water solution containing an excess of NaCN were added. The resulting mixture was filtered through Celites. The organic phase was washed with two portions of water and dried over Na_2_SO_4_. The product was purified by column chromatography (Silica gel, eluted with DCM, R_f_ = 0.35). Off-white powder, yield 332 mg (79%). ^1^H NMR (400 MHz, acetone-d_6_): *δ* 8.74 (d, *J* = 2.1 Hz, 2H), 8.47 (d, *J* = 8.3 Hz, 2H), 8.00 (dd, *J* = 8.2, 2.2 Hz, 2H), 0.30 (s, 18H). ESI HRMS [M+H]^+^ calcd. C_20_H_25_N_2_Si_2_ 349.1551, found 349.1552, [M+Na]^+^ calcd. C_20_H_24_N_2_Si_2_Na 371.1370, found 371.1392.

**4,4’-trimethylsilylethynyl-2,2’-bipyridine, 2^P^.** The same procedure as for **1^P^**. Product was purified by column chromatography (Silica gel, eluted with DCM, then EtOAc/hexane/DCM. R_f_ (EtOAc/hexane/DCM) = 0.75). Off-white powder, yield 296 mg (71%). ^1^H NMR (400 MHz, acetone-d_6_): δ 8.71 (d, *J* = 4.9 Hz, 2H), 8.46 (m, 2H), 7.46 (dd, *J* = 4.8, 1.7 Hz, 2H), 0.31 (s, 18H). ESI HRMS (m/z): calcd. For [M+H]^+^ calcd. C_20_H_25_N_2_Si_2_ 349.1551, found 349.1539.

**3,8-trimethylsilylethynyl-1,10-phenanthroline, 3^P^.** The same procedure as for **1^P^**. Product was purified by column chromatography (Silica gel, eluted with DCM/hexane 1:1, then EtOAc/hexane/DCM. R_f_ (EtOAc/hexane/DCM) = 0.85). White solid, yield 86 mg (21 %). ^1^H NMR (400 MHz, acetone-d_6_) δ 9.11 (m, 2H), 8.55 (d, *J* = 2.1 Hz, 2H), 8.02 (s, 2H), 0.33 (s, 18H). ESI HRMS [M+H]^+^ calcd. C_22_H_25_N_2_Si_2_ 373.1551, found 373.1464, [M+Na]^+^ calcd. C_22_H_24_N_2_Si_2_Na 395.1370, found 395.1359.

**Re1^P^**. Pentacarbonylrhenium(I) chloride (50 mg, 0.14 mmol) and **1^P^** (1 eq) were suspended in toluene (15 mL) and degassed by purging nitrogen for 15 min upon stirring. Then, the reaction mixture was refluxed for 3 h under an argon atmosphere to give a dark orange solution. The solvent was removed by rotary evaporation to give an oil. The product was dissolved in dichloromethane and precipitated in hexane, the solid obtained was washed with pentane and dried. Orange solid, yield 78 mg (85%). ^1^H NMR (400 MHz, chloroform-d): δ 9.08 (dd, *J* = 1.8, 0.8 Hz, 2H), 8.09 – 8.01 (m, 4H), 0.31 (s, 18H). ESI^+^ MS (m/z) 619.08 [M – Cl]^+^ (calcd. C_23_H_24_N_2_O_3_ReSi_2_ 619.08), 647.09 [M – Cl + N_2_]^+^ (calcd. C_23_H_24_N_4_O_3_ReSi_2_ 647.09), 677.02 [M + Na]^+^ (calcd. C_23_H_24_ClN_2_NaO_3_ReSi_2_ 677.04).

**Re2^P^**. Pentacarbonylrhenium(I) chloride (50 mg, 0.14 mmol) and **2^P^** (50 mg, 0.14 mmol) were suspended in toluene (15 mL) and degassed by purging nitrogen for 15 min upon stirring. Then the reaction mixture was refluxed for 3 h under an argon atmosphere to give an orange suspension. The precipitate was collected, washed with diethyl ether, and dried. Orange solid, yield 86 mg (96%). ^1^H NMR (400 MHz, acetone-d_6_)^:^ δ 9.44 (d, J = 1.8 Hz, 2H), 8.99 (d, *J* = 1.8 Hz, 2H), 8.32 (s, 2H), 0.35(s, 18H). ESI^+^ MS (m/z) 619.08 [M – Cl]^+^ (calcd. C_23_H_24_N_2_O_3_ReSi_2_ 619.08), 647.09 [M – Cl + N_2_]^+^ (calcd. C_23_H_24_N_4_O_3_ReSi_2_ 647.09), 677.03 [M + Na]^+^ (calcd. C_23_H_24_ClN_2_NaO_3_ReSi_2_ 677.04).

**Re3^P^**. The same procedure as for **Re1^P^**. Orange solid, yield 88 mg (94%). ^1^H NMR (400 MHz, chloroform-d): δ 8.95 (d, *J* = 1.8 Hz, 2H), 8.14 (bs, 2H), 8.47 (dd, *J* = 5.7, 1.6 Hz, 2H), 0.35 (s, 18H). ESI^+^ MS (m/z) 643.07 [M – Cl]^+^ (calcd. C_25_H_24_N_2_O_3_ReSi_2_ 643.08), 671.09 [M – Cl + N_2_]^+^ (calcd. C_25_H_24_N_4_O_3_ReSi_2_ 671.09), 701.03 [M + Na]^+^ (calcd. C_25_H_24_ClN_2_NaO_3_ReSi_2_ 701.04).

**General procedure for the synthesis of complexes ReX.** To a stirred suspension of **ReX^P^** (40 mg, 0.06 mmol) in MeOH (15 mL) K_2_CO_3_ (25 mg, 0.018 mmol) was added. The reaction mixture was stirred for 4 h, and then the solvent was removed by rotary evaporation. The product was dissolved in dichloromethane and washed with water, the organic layer was separated and dried over MgSO_4_. The solvent was removed to a minimum by rotary evaporation and the residue was precipitated in hexane. The precipitate was collected, washed with diethyl ether and dried. 

**Re1**, orange solid, yield 40 mg (80%) ^1^H NMR (400 MHz, acetone-d_6_): δ 9.16 (d, *J* = 1.9 Hz, 2H), 8.76 (d, *J* = 8.5 Hz, 2H), 8.42 (dd, *J* = 8.5, 2.0 Hz, 2H), 4.34 (s, 2H). ESI^+^ MS (m/z) 475.00 [M – Cl]^+^ (calcd. C_17_H_8_N_2_O_3_Re 475.01), 503.01 [M – Cl + N_2_]^+^ (calcd. C_17_H_8_N_4_O_3_Re 503.01), 532.96 [M + Na]^+^ (calcd. C_17_H_8_ClN_2_NaO_3_Re 532.96).

**Re2**, orange solid, yield 66 mg (84%).^1^H NMR (400 MHz, chloroform-d): δ 9.04 (d, *J* = 5.8, 2H), 8.22 (s, 2H), 7.58 (dd, *J* = 5.7, 1.6 Hz, 2H), 3.67 (s, 2H). ESI^+^ MS (m/z) 475.00 [M – Cl]^+^ (calcd. C_17_H_8_N_2_O_3_Re 475.01), 503.01 [M – Cl + N_2_]^+^ (calcd. C_17_H_8_N_4_O_3_Re 503.01), 532.95 [M + Na]^+^ (calcd. C_17_H_8_ClN_2_NaO_3_Re 532.96).

**Re3**, orange solid, yield 55 mg (87%). ^1^H NMR (400 MHz, acetone-d_6_) δ 9.50 (s, 2H), 9.07 (s, 2H), 8.36 (s, 2H), 4.39 (s, 2H). ESI^+^ MS (m/z) 499.01 [M – Cl]^+^ (calcd. C_19_H_8_N_2_O_3_Re 499.01), 527.01 [M – Cl + N_2_]^+^ (calcd. C_19_H_8_N_4_O_3_Re 527.01), 556.96 [M + Na]^+^ (calcd. C_19_H_8_ClN_2_NaO_3_Re 556.96).

**General procedure for the synthesis of complexes ReX^C^. ReX** (15 mg, 0.03 mmol) was dissolved in acetone (8 mL) and 2-azidomethylpyridine was added. To the stirring mixture, an aqueous solution (2 mL) of CuSO_4_×5H_2_O (8 mg, 0.03 mmol) и NaAsc (12 mg, 0.06 mmol) was added. The reaction mixture was stirred for 12 h and then the solvent was removed. The crude product was dissolved in dichloromethane (30 mL) and washed with NH_4_OH/EDTA solution. The organic layer was separated and dried over MgSO_4_ and the solvent was evaporated. The product was dissolved in dichloromethane and precipitated in hexane. The precipitate was collected, washed with diethyl ether and dried.

**Re1^C^**, yellow solid, 19 mg (85%). ^1^H NMR (400 MHz, acetone-d_6_): δ 9.61 (s, 1H), 8.93 (s, 1H), 8.75 (d, *J* = 2.5 Hz, 2H), 8.59 (d, *J* = 4.6 Hz, 1H), 7.86 (d, *J* = 1.8 Hz, 1H), 7.44 (d, *J* = 7.8 Hz, 1H), 7.41 – 7.35 (m, 1H), 5.89 (s, 2H). ESI^+^ MS (m/z) 743.12 [M – Cl]^+^ (calcd. C_29_H_20_N_10_O_3_Re 743.13), 771.13 [M – Cl + N_2_]^+^ (calcd. C_29_H_20_N_12_O_3_Re 771.13), 801.08 [M + Na]^+^ (calcd. C_29_H_20_ClN_10_NaO_3_Re 801.08).

**Re2^C^**, yellow solid, 21 mg (70%). ^1^H NMR (400 MHz, chloroform-d): δ 8.92 (s, 2H), 8.73 (d, *J* = 5.8 Hz, 1H), 8.69 (d, *J* = 3.7 Hz, 1H), 8.61 (s, 2H), 7.81 (td, *J* = 7.8, 1.8 Hz, 2H), 7.71 (d, *J* = 5.8 Hz, 2H), 7.41 (d, *J* = 7.9 Hz, 2H), 7.34 (dd, *J* = 7.6, 4.9 Hz, 2H), 5.89 – 5.74 (m, 4H). ESI^+^ MS (m/z) 743.12 [M – Cl]^+^ (calcd. C_29_H_20_N_10_O_3_Re 743.12), 771.12 [M – Cl + N_2_]^+^ (calcd. C_29_H_20_N_12_O_3_Re 771.13), 801.07 [M + Na]^+^ (calcd. C_29_H_20_ClN_10_NaO_3_Re 801.08).

**Re3^C^**. Yellow solid, yield 53 mg (70%). ^1^H NMR (400 MHz, acetone-d_6_) δ 9.97 (d, *J* = 1.7 Hz, 1H), 9.30 (s, 1H), 9.04 (s, 1H), 8.61 (d, *J* = 4.9 Hz, 1H), 8.32 (s, 1H), 7.87 (td, *J* = 7.7, 1.8 Hz, 1H), 7.47 (d, *J* = 7.9 Hz, 1H), 7.39 (dd, *J* = 7.4, 4.9 Hz, 1H), 5.92 (s, 2H). ESI^+^ MS (m/z) 808.15 [M – Cl + CH_3_CN]^+^ (calcd. C_33_H_23_N_11_O_3_Re 808.15), 820.13 [M – Cl + CH_2_ONa]^+^ (calcd. C_32_H_22_N_10_NaO_4_Re 820.13).

**General procedure for the synthesis of complexes Ir(C^N)X^P^**. 2.1 eqv. **1^P^** or **2^P^** was added to **[Ir(C^N)_2_Cl]_2_** and suspended in a mixture of CH_2_Cl_2_/MeOH (3:1), followed by the addition of an excess of KPF_6_. The reaction mixture was stirred for 24h at room temperature. After filtration through the Celite the resulting solution was evaporated to dryness. A small amount of CH_2_Cl_2_ was added, then a solid was precipitated with hexane. The precipitate was washed with diethyl ether twice.

**Ir(ppy)1^P^.** Dark-red powder. Yield: 50.6 mg (64%). ^1^H NMR (400 MHz, Acetone-d_6_): δ 8.87 (d, *J* = 8.5 Hz, 2H), 8.34 – 8.24 (m, 4H), 8.07 – 7.90 (m, 10H), 7.23 – 7.15 (m, 2H), 7.08 (td, *J* = 7.5, 1.3 Hz, 2H), 6.97 (td, *J* = 7.4, 1.3 Hz, 2H), 6.35 (d, *J* = 7.2 Hz, 2H), 0.20 (s, 18H). ESI^+^ MS (m/z) 849.24 [M – PF_6_]^+^ (calcd. C_42_H_40_IrN_4_Si_2_ 849.24).

**Ir(dfppy)1^P^.** Dark-yellow powder. Yield: 54.3 mg (54%). ^1^H NMR (400 MHz, Acetone-d_6_): δ 8.91 (d, *J* = 8.4 Hz, 2H), 8.45 – 8.39 (m, 2H), 8.38 – 8.33 (m, 2H), 8.14 – 8.03 (m, 6H), 7.31 – 7.24 (m, 2H), 6.85 – 6.75 (m, 2H), 5.80 (dd, J = 8.5, 2.3 Hz, 2H), 0.21 (s, 18H). ESI^+^ MS (m/z) 921.20 [M – PF_6_]^+^ (calcd. C_42_H_36_F_4_IrN_4_Si_2_ 921.20).

**Ir(ppy)2^P^.** Orange powder. Yield: 41.8 mg (53%). ^1^H NMR (400 MHz, Acetone-d_6_): δ 8.97 (s, 2H), 8.26 (d, *J* = 8.1 Hz, 2H), 8.09 (d, *J* = 5.7 Hz, 2H), 7.99 (t, *J* = 7.7 Hz, 2H), 7.91 (d, *J* = 6.8 Hz, 4H), 7.71 (d, *J* = 5.6 Hz, 2H), 7.19 (t, *J* = 6.5 Hz, 2H), 7.06 (t, *J* = 7.5 Hz, 2H), 6.94 (t, *J* = 7.4 Hz, 2H), 6.34 (d, *J* = 7.5 Hz, 2H), 0.30 (s, 18H).

**Ir(dfppy)2^P^.** Orange powder. Yield: 60.5 mg (61%). ^1^H NMR (400 MHz, Acetone-d_6_): δ 9.00 (d, *J* = 1.0 Hz, 2H), 8.47 – 8.36 (m, 2H), 8.23 – 8.17 (m, 2H), 8.14 – 8.06 (m, 2H), 8.05 – 7.96 (m, 2H), 7.73 (dd, *J* = 5.8, 1.7 Hz, 2H), 7.28 (ddd, *J* = 7.5, 5.8, 1.4 Hz, 2H), 6.78 (ddd, *J* = 12.8, 9.3, 2.4 Hz, 2H), 5.79 (dd, *J* = 8.5, 2.4 Hz, 2H), 0.30 (s, 18H). ESI^+^ MS (m/z) 921.20 [M – PF_6_]^+^ (calcd. C_42_H_36_F_4_IrN_4_Si_2_ 921.20).

**General procedure for the synthesis of complexes Ir(C^N)X. Ir(C^N)X^P^** (except **Ir(dfppy)2^P^** where 1.2 eqv. KOH was added) was dissolved in MeOH and stirred overnight, then filtered through a pad of Celite and the solvent was evaporated to obtain the powder product.

**Ir(ppy)1.** Dark-orange powder. Yield: 45 mg (89%). ^1^H NMR (400 MHz, Acetone-d_6_): δ 8.90 (d, *J* = 8.5 Hz, 2H), 8.37 (d, *J* = 8.5 Hz, 2H), 8.27 (d, *J* = 8.2 Hz, 2H), 8.11 (s, 2H), 8.04 – 7.96 (m, 4H), 7.94 (d, *J* = 7.7 Hz, 2H), 7.18 (t, *J* = 6.7 Hz, 2H), 7.08 (t, *J* = 7.6 Hz, 2H), 6.97 (t, *J* = 7.5 Hz, 2H), 6.37 (d, *J* = 7.5 Hz, 2H), 4.20 (s, 2H). ESI^+^ MS (m/z) 705.16 [M – PF_6_]^+^ (calcd. C_36_H_24_IrN_4_ 705.16).

**Ir(dfppy)1.** Orange powder. Yield: 30 mg (64%). ^1^H NMR (400 MHz, Acetone-d_6_): δ 8.94 (d, *J* = 8.5 Hz, 2H), 8.42 (dd, *J* = 8.5, 2.1 Hz, 4H), 8.20 (d, *J* = 1.9 Hz, 2H), 8.13 – 8.05 (m, 4H), 7.26 (ddd, *J* = 7.4, 5.8, 1.4 Hz, 2H), 6.80 (ddd, *J* = 12.8, 9.4, 2.4 Hz, 2H), 5.81 (dd, *J* = 8.5, 2.4 Hz, 2H), 4.23 (s, 2H). ESI MS (m/z) 777.12 [M – PF_6_]^+^ (calcd. C_36_H_20_F_4_IrN_4_ 777.13).

**Ir(ppy)2.** Orange powder. Yield: 34.4 mg (82%). ^1^H NMR (400 MHz, Acetone-d_6_): δ 9.04 (s, 2H), 8.26 (d, *J* = 8.2 Hz, 2H), 8.12 (d, *J* = 5.6 Hz, 2H), 7.99 (td, *J* = 7.8, 1.5 Hz, 2H), 7.96 – 7.89 (m, 4H), 7.77 (dd, *J* = 5.7, 1.7 Hz, 2H), 7.23 – 7.14 (m, 2H), 7.06 (td, *J* = 7.5, 1.3 Hz, 2H), 6.94 (td, *J* = 7.4, 1.3 Hz, 2H), 6.34 (dd, *J* = 7.5, 1.2 Hz, 2H), 4.47 (s, 2H). ESI^+^ MS (m/z) 705.16 [M – PF_6_]^+^ (calcd. C_36_H_24_IrN_4_ 705.16).

**Ir(dfppy)2.** Dark-yellow powder. Yield: 28.4 mg (47%). ^1^H NMR (400 MHz, Acetone-d_6_): δ 8.77 (d, *J* = 1.7 Hz, 2H), 8.41 (d, *J* = 8.3 Hz, 2H), 8.09 (d, *J* = 5.9 Hz, 4H), 7.95 – 7.83 (m, 2H), 7.67 (dd, *J* = 5.7, 1.7 Hz, 2H), 7.26 (ddd, *J* = 7.4, 5.8, 1.4 Hz, 2H), 6.84 – 6.69 (m, 2H), 5.87 – 5.73 (m, 2H), 4.73 (t, *J* = 5.3 Hz, 2H). 

**General procedure for the synthesis of complexes Ir(C^N)X^C^. Ir(C^N)X** (1 eqv.) was dissolved in 12 mL of acetone and 2-(azidomethyl)pyridine (2.3 eqv.) was added. CuSO_4_×5H_2_O (2 eqv.) and sodium ascorbate (4 eqv.) were suspended in 3 mL of water and added to the solution. The reaction mixture was stirred for 48h at room temperature. Water and an aqueous solution of NH_4_OH/EDTA were added to the resulting solution and stirred for 30 min. The organic phase was collected, dried over MgSO_4,_ and evaporated to give an oil. It was dissolved in a small amount of DCM and hexane was added to precipitate a solid. The product was washed with diethyl ether and dried *in vacuo*.

**Ir(ppy)1^C^.** Orange powder. Yield: 15.1 mg (45%). ^1^H NMR (400 MHz, Acetone-d_6_): δ 8.91 (d, *J* = 8.5 Hz, 2H), 8.68 (d, *J* = 2.0 Hz, 2H), 8.65 (dd, *J* = 8.4, 2.1 Hz, 2H), 8.57 (d, *J* = 4.5 Hz, 2H), 8.46 (s, 2H), 8.24 (d, *J* = 8.2 Hz, 2H), 8.04 (d, *J* = 5.9 Hz, 2H), 8.00 – 7.89 (m, 4H), 7.85 (td, *J* = 7.8, 1.9 Hz, 2H), 7.43 (d, *J* = 7.8 Hz, 2H), 7.38 (dd, *J* = 7.7, 4.9 Hz, 2H), 7.19 – 7.12 (m, 2H), 7.11 – 7.05 (m, 2H), 6.97 (td, *J* = 7.4, 1.1 Hz, 2H), 6.42 (d, *J* = 7.4 Hz, 2H), 5.78 (s, 4H), ESI^+^ MS (m/z) 973.29 [M – PF_6_]^+^ (calcd. C_48_H_36_IrN_12_ 973.28).

**Ir(dfppy)1^C^.** Orange powder. Yield: 23.2 mg (72%). ^1^H NMR (400 MHz, Acetone-d_6_): δ 8.98 – 8.93 (m, 2H), 8.73 – 8.68 (m, 4H), 8.59 (s, 2H), 8.56 (d, *J* = 4.4 Hz, 2H), 8.42 – 8.38 (m, 2H), 8.13 (d, *J* = 5.2 Hz, 2H), 8.06 (t, *J* = 7.8 Hz, 3H), 7.85 (td, *J* = 7.7, 1.7 Hz, 2H), 7.43 (d, *J* = 7.8 Hz, 2H), 7.40 – 7.35 (m, 2H), 7.24 (t, *J* = 6.1 Hz, 2H), 6.85 – 6.78 (m, 2H), 5.87 (dd, *J* = 8.5, 2.3 Hz, 2H), 5.78 (s, 4H). ESI^+^ MS (m/z) 1045.24 [M – PF_6_]^+^ (calcd. C_48_H_32_F_4_IrN_12_ 1045.24).

**Ir(ppy)2^C^.** Orange powder. Yield: 9.1 mg (48%). ^1^H NMR (400 MHz, Acetone-d_6_): δ 9.38 (s, 2H), 9.00 (s, 2H), 8.60 – 8.55 (m, 2H), 8.26 (d, *J* = 8.1 Hz, 2H), 8.18 – 8.14 (m, 2H), 8.14 – 8.11 (m, 2H), 8.02 – 7.96 (m, 4H), 7.94 – 7.91 (m, 2H), 7.87 (td, *J* = 7.7, 1.7 Hz, 2H), 7.48 (d, *J* = 7.8 Hz, 2H), 7.41 – 7.36 (m, 2H), 7.17 (t, *J* = 6.6 Hz, 2H), 7.06 (t, *J* = 7.5 Hz, 2H), 6.95 (t, *J* = 6.9 Hz, 2H), 6.39 (d, *J* = 7.5 Hz, 2H), 5.88 (s, 4H). ESI^+^ MS (m/z) 973.28 [M – PF_6_]^+^ (calcd. C_48_H_36_IrN_12_ 973.28).

**Pt1^P^.** This complex was prepared according to the literature methods [19]. Brownish powder, yield 22.7 mg (62%). ^1^H NMR (400 MHz, Chloroform-*d*) δ 9.82 (m, 2H, dtbpy), 8.13 (t, *J* = 8.0 Hz, 2H, dtbpy), 8.05 (d, *J* = 8.5 Hz, 2H, dtbpy), 7.56 (m, 6H, Ph), 7.32 (m, 6H, Ph), 7.20 (t, *J* = 7.5 Hz, 4H, Ph), 3.75 (s, 2H, alkynyl-H). ESI^+^ MS (m/z) 640.07 [M + K]^+^ (calcd. C_30_H_18_KN_2_Pt 640.07).

**Pt(dtbpy)^C^.** Pt(dtbpy)(C≡CPh)_2_ (12.6 mg, 0.019 mmol) and 2-azidomethylpyridine (5 mg, 0.047 mmol) were dissolved in 20 mL of CH_2_Cl_2_. 3 eq CuSO_4_×5H_2_O (14 mg, 0.057 mmol) and NaAsc (22.5 mg, 0.114 mmol) were dissolved in 5 mL of MeOH, sonicated for 3 min and added to the acetone solution. The reaction mixture was flushed with argon and stirred for 48 h at 40 °C. The solvents were removed in vacuo, and 20 mL of CHCl_3_, 10 mL of water and 2 mL of 5% EDTA solution were added to the residue. The resulting mixture was vigorously stirred for 30 min, then the organic layer was separated, and the solvent was removed in vacuo to a minimal volume. Then, diethyl ether was added to obtain a crude product as a yellow solid. The resulting powder was washed with diethyl ether, dried in vacuo and recrystallized from an acetone solution. Yellow crystals, yield 12 mg (69%). ^1^H NMR (400 MHz, DMSO-d_6_): δ 8.54 (d, *J* = 1.8 Hz, 2H, dtbpy), 8.36 (m, 4H, i-click (o-Ph)), 8.11 (m, 2H, i-click (py)), 7.89 (d, *J* = 6.0 Hz, 2H, dtbpy (H3, H4)), 7.49 (dd, *J* = 6.0 Hz, 1.8 Hz, 2H, dtbpy (H2, H5)), 7.44 (td, *J* = 7.7 Hz, 1.8 Hz, 2H, i-click (py)), 7.12 (m, 4H, i-click (m-Ph)), 7.07 (m, 2H, i-click (p-Ph)), 7.03 (m, 2H, i-click (py)), 6.64 (d, *J* = 7.9 Hz, 2H, i-click (py)), 5.36 (d, *J* = 15.8 Hz, 2H, CH_2_), 5.36 (d, *J* = 15.8 Hz, 2H, CH_2_), 1.35 (s, 21H, ^t^Bu). ESI^+^ MS (m/z) 934.36 [M + H]^+^ (calcd. C_46_H_46_N_10_Pt 934.36). Single crystals of **Pt(dtbpy)^C^** were obtained by slow evaporation of an acetone solution at room temperature. 

### 4.3. X-ray Crystal Structure Determination

The crystal structure of **Pt(dtbpy)^C^** was determined by the means of single crystal XRD analysis using a Rigaku Oxford Diffraction XtaLAB HyPix-3000 diffractometer (Rigaku Oxford Diffraction, Oxford, UK) for the data collection at a temperature of 170K. Diffraction data were processed in the *CrysAlisPro* program (Agilent Technologies, Version 1.171.39.35a, Stockport, UK) [80]. The unit-cell and refinement parameters are listed in Table S1. The structures were solved by the dual-space algorithm and refined using the SHELX programs [81,82] incorporated in the OLEX2 program package [83]. Supplementary crystallographic data for this paper have been deposited at Cambridge Crystallographic Data Centre and available online: https://www.ccdc.cam.ac.uk/structures/ (accessed on 14 August 2022). **Pt(dtpby)c**: (P2_1_/n (14), *a* = 11.56500(10), *b* = 20.6552(2), *c* = 18.3102(2) Å; *α* = 90, *β* = 99.1890(10), *γ* = 90; *V* = 4317.76(7) Å^3^; *Z* = 4; *R*_1_ = 0.0247%; CCDC 2204302). 

## 5. Conclusions

In the present study, we applied the CuAAC reaction for the post-synthetic modification of diimine mononuclear complexes Re(I), Pt(II) and Ir(III) with terminal C≡C bonds at the periphery of the ligand environment and demonstrated the efficiency of the synthetic approach for different metallocentres. All functionalized complexes obtained were characterized by spectroscopic methods. It was found that the position of C≡C bonds in the aromatic ring of the diimine ligand and the composition of the ligand environment forming the first coordination sphere can influence the post-synthetic modification of Ir(III) complexes. The implementation of the standard click protocol for Pt(II) bis-alkynyl complexes bearing the same diimine ligands did not result in the formation of the corresponding click products clearly demonstrating competition between the terminal and organoplatinum acetylene units in the click process. While replacing the diinine with “click-innocent” one, the reaction occurs as a platinum iclick process and bis-triazolyl derivative was obtained in good yield. It was found that post-synthetic modification of the far periphery of the diimino ligand in Re(I) and Ir(III) complexes by click-reaction can change the luminescence properties of the metallocentre, which should be considered when designing molecular systems that exploit the emission properties of the complex. Thus, the data obtained in the present study, together with literature data, show that click-reaction has a high potential for the construction of complex molecular systems involving transition metal complexes and can be used as a powerful tool for the rational molecular design of smart functional materials. 

## Data Availability

All data reported herein are accompanying the present article.

## Supplementary Materials

The following supporting information can be downloaded at: https://www.mdpi.com/1420-3049/27/19/6494/s1, X-ray structure determination; Table S1: Crystallographic data for compound Pt(dtbpy)^C^; Figure S1: ^1^H NMR spectra of **NN** compounds **XP** (X = 1−3); Figure S2: ^1^H NMR spectra of **Re1^X^** complexes; Figure S3: ^1^H NMR spectra of **Re2^X^** complexes; Figure S4: ^1^H NMR spectra of **Re3^X^** complexes; Figure S5: ESI^+^ MS spectra of **Re1^X^** complexes with isotope pattern of key signals; Figure S6: ESI^+^ MS spectra of **Re2^X^** complexes with isotope pattern of key signals; Figure S7: ESI^+^ MS spectra of **Re3^X^** complexes with isotope pattern of key signals; Figure S8: ^1^H NMR spectra of **Ir(ppy)1^X^** complexes; Figure S9: ^1^H NMR spectra of **Ir(ppy)2^X^** complexes; Figure S10: ^1^H NMR spectra of **Ir(dfppy)1^X^** complexes; Figure S11: ^1^H NMR spectra of **Ir(dfppy)2^X^** complexes; Figure S12: ESI^+^ MS spectra of **Ir(ppy)1^X^** complexes with isotope pattern of key signals; Figure S13: ESI^+^ MS spectra of **Ir(ppy)2^X^** complexes with isotope pattern of key signals; Figure S14: ESI^+^ MS spectra of **Ir(dfppy)1^X^** complexes with isotope pattern of key signals; Figure S15: ESI^+^ MS spectra of **Ir(dfppy)2^P^** complexes with isotope pattern of key signals; Figure S16: ^1^H NMR spectra of **Pt1^X^** complexes; Figure S17: ^1^H NMR spectra of **Pt2^X^** complexes; Figure S18: ^1^H NMR spectrum of **Pt(btbpy)^C^** complexes in aromatic region, DMSO-d_6_, RT; Figure S19: ^1^H^1^H COSY NMR spectrum of **Pt(btbpy)^C^** complexes in aromatic region, DMSO-d_6_, RT; Figure S20: ESI^+^ MS spectrum of **Pt(dtbpy)^C^** complex with isotope pattern of key signals; Figure S21: Molecular structure of **Pt(dtbpy)^C^** complex with key atoms numeration; Table S2: Selected structural parameters for compound **Pt(dtbpy)^C^** and literature data for Pt(II) triazolyl derivatives; Table S3: Photophysical properties of “clicked” complexes **ReX^C^** and **IrX^C^**.

## Sample Availability

Samples of all compounds described in the article are available from the authors.

## Appendix A

**Table A1:** Some literature examples of C≡C peripherally functionalized metallocenters, azide substrates and click-reaction conditions.

##	Metal	Substrate	Conditions; yield	Reference
1	Re(I), Mo(I)		Cu(SO_4_)_2_ × 5H_2_O, NaAsc, THF/H_2_O, r.t., 120 h; up to 88%	[6]
2	Re(I), Tc(I)		Cu(MeCOO)_2_, PBS, NaAsc, ^t^BuOH/H_2_O, r.t., 1.5 h; 2–84%	[7]
3			Cu(MeCOO)_2_, NaAsc, ^t^BuOH/H_2_O, 70 °C, 1.5 h; 81%	[8]
4	Re(V), Tc(V)	azido-modified angiotensin-II peptide	Cu(SO_4_)_2_ × 5H_2_O, NaAsc, CH_2_Cl_2_/MeOH/H_2_O, r.t., 5 h; 51%	[9]
5	Re(I)		Cu(SO_4_)_2_ × 5H_2_O, NaAsc, DMF/H_2_O, r.t., 24 h; 84%	[10]
6			Cu(SO_4_)_2_ × 5H_2_O, NaAsc, THF/H_2_O, 60 °C, 18 h; 93%	[11]
7	Ir(III)		Cu(SO_4_)_2_ × 5H_2_O, NaAsc, DMF/H_2_O, r.t., 12 h; 71–88%	[12]
8		Au(PR_3_)N_3_	DIPEA, DMF, r.t., 48 h; 20%	[13]
9			CuBr, MeCN, 70 °C, 12 h	[14]
10			CuI, ^i^Pr_2_EtN, MeCN/MeOH/HCl, r.t., 12 h	[15]
11	Pt(II)		Cu(SO_4_)_2_ ×5H_2_O, NaAsc, DMF/H_2_O, r.t., up to 16 h; 62–81%	[16,17]
12			Cu(SO_4_)_2_ × 5H_2_O, Hasc, DMF/H_2_O, r.t., 15 h; 62–54%	[18]
13			Cu(MeCN)_4_PF_6_, Cu, DIPEA, CH_2_Cl_2_/MeOH, r.t., 72 h; 73–84%	[19]
14			Cu(SO_4_)_2_ × 5H_2_O, Hasc, CH_2_Cl_2_/H_2_O, reflux, 48 h; 75%	[20]
15			Cu(MeCN)_4_PF_6_, Cu, DIPEA, CH_2_Cl_2_/MeOH, r.t., up to 192 h; 17-84%	[21]
16			Cu(MeCN)_4_PF_6_, Cu, Et(^i^Pr)_2_N, CH_2_Cl_2_/MeOH, reflux, 312 h; 15%	[22]
17	Pt(IV)	azido-functionalized enterobactin	Cu(MeCN)_4_PF_6_, TBTA, DMF/H_2_O, r.t., 3 h	[24]
18	Ru(II, III)		Cu(SO_4_)_2_ × 5H_2_O, NaAsc, ^t^BuOH/H_2_O, r.t., 12 h; 63%	[25]
19		azido-containing poly(benzylether) dendrons	Cu(SO_4_)_2_ × 5H_2_O, NaAsc, THF/H_2_O, r.t., 12 h; ca. 50%	[26,27]
20	Ru(II)		Cu(SO_4_)_2_ × 5H_2_O, NaAsc, (^i^Pr)_2_EtN, ^t^BuOH/MeOH/H_2_O, r.t., 18 h; 80%	[28]
21			Cu(SO_4_)_2_ × 5H_2_O, NaAsc, CH_2_Cl_2_/H_2_O, r.t., 20 h; 58–81%	[29]
22			Cu(SO_4_)_2_ × 5H_2_O, NaAsc, CH_2_Cl_2_/H_2_O, r.t., 12 h; 67–69%	[30]
23			Cu(SO_4_)_2_ × 5H_2_O, NaAsc, CH_2_Cl_2_/H_2_O, r.t., 20 h; 61%	[31]
24			Cu(SO_4_)_2_ × 5H_2_O, NaAsc, acetone/H_2_O, r.t., 1h; quantitative	[32]
25			Cu(SO_4_)_2_ × 5H_2_O, NaAsc, CH_2_Cl_2_/H_2_O, r.t., 48 h; 55%	[33]
26	Fe(II)		Cu(SO_4_)_2_ × 5H_2_O, NaAsc, CH_2_Cl_2_/NCMe/H_2_O, r.t., 12 h; 79%	[35]
27			Cu(MeCOO)_2_, toluene, 70 °C, 2 h; 67%	[36]
28	ferrocene		Cu(SO_4_)_2_ × 5H_2_O, NaAsc, THF/H_2_O, r.t, 12 h; up to 84%	[37]
29		azido-functionalized siloxanes	various	[38]
30			various	[39]
31			Cu(SO_4_)_2_×5H_2_O, NaAsc, MeOH/H_2_O, TTTA, r.t., 20 h; 17-86%	[40]
32			Cu(SO_4_)_2_×5H_2_O, NaAsc, THF/H_2_O, 60°C, 6 h; 69%	[41]
			Cu(SO_4_)_2_×5H_2_O, NaAsc, THF/H_2_O, r.t., 24 h; 75%	
33	Mn(I)		Cu(SO_4_)_2_×5H_2_O, NaAsc, ^t^BuOH/ H_2_O, r.t., 24 h; 61%	[42]
34	Ni(II)		CuI, Et_3_N, DMSO, 70°C, 5 h; 31-92%	[43]
35	Zn-porphyrin		CuI, DBU, toluene, 70°C, 4 h; 90%	[44]
36			CuI, DIPEA, CHCl_3_, reflux, 12 h; 87%	[45]
37	Cr(0), W(0)	various	various	[48]
38	Au(I)	azido-amphiphilic oligopeptide	Cu(SO_4_)_2_×5H_2_O, NaAsc, TBTA, THF/H_2_O, 24 h, 45°C; up to 87%	[49]
39	Tb(III)		Cu(SO_4_)_2_×5H_2_O, NaAsc, THPTA, H_2_O/MeCN, r.t., 12 h, 37%	[50]
40	Ln(III)	various, including azido-functionalized Ln(III) complexes	various	[51,52,53,54,55,56]
41	{MC_3_B_7_H_9_}, M = Fe, Ru		Cu(SO_4_)_2_×5H_2_O, NaAsc, ^t^BuOH/H_2_O, r.t., up to 19 h; up to 82%	[57]
42	M-bis(1,2-dicarbollide); M = Fe, Co	azido-cholesterol	CuI, DIPEA, EtOH, reflux, 12 h	[58]
43	POM		CuI, DIPEA, MeCN, r.t., 3 h; up to 97%	[59]
44		Au(PR_3_)N_3_	DIPEA, DMF, 35°C, 50 h; 60%	[60]

NaAsc = sodium ascorbate. DIPEA = diisopropylethylamin. DBU = 1,8-diazabicyclo[5 .4.0]undec-7-ene. TTTA = tris(1-tert-butyl-1H-1,2,3-triazolyl)methyl amine. POM = polyoxometalate. TBTA = tris(benzyltriazolylmethyl)amine. THPTA = tris(hydroxypropyltriazolylmethyl)amine.
