# Supplementary material for: Post-Functionalization of Organometallic Complexes via Click-Reaction
Source: Molecules. 2022 Oct 1;27(19):6494. doi: 10.3390/molecules27196494 (PMC9614606; doi:10.3390/molecules27196494)
Supplement: Supplementary file 1 [file molecules-27-06494-s001.zip › molecules-1916790-supplementary-v6.pdf]

**Supplementary Information**

# Post-functionalization of organometallic complexes *via* click-reaction

Stanislav Petrovskii, Viktoria Khistiaeva, Aleksandra Paderina, Evgenia Abramova and Elena Grachova *

Institute of Chemistry, St Petersburg University, 198504 St. Petersburg, Russia

Correspondence: e.grachova@spbu.ru

**Content**


Content 2

X-ray structure determination....4

**Table S1.** Crystallographic data for compound **Pt(dtbpy)**$^{C}$. ....5

**Figure S1.** $^{1}$H NMR spectra of **NN** compounds **XP** (X = 1−3). Aromatic (left) and aliphatic (right) regions are shown on a different vertical scale. ....6

**Figure S2.** $^{1}$H NMR spectra of **Re1**$^{X}$ complexes. The residual solvent peak of $CDCl_3$ is marked by asterisk. ....7

**Figure S3.** $^{1}$H NMR spectra of **Re2**$^{X}$ complexes. The residual solvent peak of $CDCl_3$ is marked by asterisk. ....8

**Figure S4.** $^{1}$H NMR spectra of **Re3**$^{X}$ complexes. The resonances of admixtures are marked by asterisks. ....9

**Figure S5.** ESI$^{+}$ MS spectra of **Re1**$^{X}$ complexes with isotope pattern of key signals. ....10

**Figure S6.** ESI$^{+}$ MS spectra of **Re2**$^{X}$ complexes with isotope pattern of key signals. ....11

**Figure S7.** ESI$^{+}$ MS spectra of **Re3**$^{X}$ complexes with isotope pattern of key signals. ....12

**Figure S8.** $^{1}$H NMR spectra of **Ir(ppy)1**$^{X}$ complexes. ....13

**Figure S9.** $^{1}$H NMR spectra of **Ir(ppy)2**$^{X}$ complexes. ....14

**Figure S10.** $^{1}$H NMR spectra of **Ir(dfppy)1**$^{X}$ complexes. ....15

**Figure S11.** $^{1}$H NMR spectra of **Ir(dfppy)2**$^{X}$ complexes. ....15

**Figure S12.** ESI$^{+}$ MS spectra of **Ir(ppy)1**$^{X}$ complexes with isotope pattern of key signals. ..16

**Figure S13.** ESI$^{+}$ MS spectra of **Ir(ppy)2**$^{X}$ complexes with isotope pattern of key signals. ..17

**Figure S14.** ESI$^{+}$ MS spectra of **Ir(dfppy)1**$^{X}$ complexes with isotope pattern of key signals.... ....18

**Figure S15.** ESI$^{+}$ MS spectra of **Ir(dfppy)2**$^{P}$ complexes with isotope pattern of key signals.... ....19

**Figure S16.** $^{1}$H NMR spectra of **Pt1**$^{X}$ complexes. Asterisks mark the resonances of admixtures and the residual solvent peak of $CDCl_3$ (7.29 ppm). ....20

**Figure S17.** $^{1}$H NMR spectra of **Pt2**$^{X}$ complexes. Asterisks mark the resonances of admixtures and the residual solvent peak of $CDCl_3$ (7.29 ppm). ....21

**Figure S18.** $^{1}H$ NMR spectrum of **Pt(btbpy)$^{C}$** complexes in aromatic region, DMSO-$d_6$, RT. . ........................................22

**Figure S19.** $^{1}H^{1}H$ COSY NMR spectrum of **Pt(btbpy)$^{C}$** complexes in aromatic region, DMSO-$d_6$, RT. ........................................22

**Figure S20.** ESI$^{+}$ MS spectrum of **Pt(dtbpy)$^{C}$** complex with isotope pattern of key signals. 23

**Figure S21.** Molecular structure of **Pt(dtbpy)$^{C}$** complex with key atoms numeration. ..........23

**Table S2.** Selected structural parameters for compound **Pt(dtbpy)$^{C}$** and literature data for Pt(II) triazolyl derivatives [5,6]. ........................................24

**Table S3.** Photophysical properties of "clicked" complexes **ReX$^{C}$** and **IrX$^{C}$**, DCE, r.t., $10^{-5}$ M. ........................................24

References ........................................25

**X-ray structure determination**

The crystal structure of **Pt(dtpby)c** was determined by the means of single crystal X-ray diffraction analysis. Crystal was fixed on a micro mount and the diffraction data have been collected on the Agilent Supernova diffractometer at a temperature of 170K using monochromated Cu $K_{\alpha}$ radiation. Data were integrated and corrected for background, Lorentz, and polarization effects. An empirical absorption correction based on spherical harmonics implemented in the SCALE3 ABSPACK algorithm was applied in *CrysAlisPro* program [1]. The unit-cell parameters (Table S1) were refined by the least-squares techniques. The structure were solved by dual-space algorithm and refined using the *SHELX* programs [2,3] incorporated in the *OLEX2* program package [4].The final model included coordinates and anisotropic displacement parameters for all non-H atoms. The carbon-bound H atoms were placed in calculated positions and were included in the refinement in the 'riding' model approximation, $U_{iso}$(H) set to 1.5$U_{eq}$(C) and C–H 0.96 Å for the $CH_3$ groups, $U_{iso}$(H) set to 1.2$U_{eq}$(C) and C–H 0.97 Å for the $CH_2$ groups, $U_{iso}$(H) set to 1.2$U_{eq}$(C) and C–H 0.93 Å for the CH groups. Supplementary crystallographic data for this paper have been deposited at Cambridge Crystallographic Data Centre (CCDC 2204302) and can be obtained free of charge via www.ccdc.cam.ac.uk/structures/.

**Table S1.** Crystallographic data for compound **Pt(dtbpy)^C^**.

| Compound | **Pt(dtbpy)**$^{C}$ |
|---|---|
| Formula | $C_{49}H_{52}N_{10}OPt$ |
| Crystal system | monoclinic |
| $a$ (Å) | 11.56500(10) |
| $b$ (Å) | 20.6552(2) |
| $c$ (Å) | 18.3102(2) |
| α (°) | 90 |
| β (°) | 99.1890(10) |
| γ (°) | 90 |
| $V$ (Å$^3$) | 4317.76(7) |
| Molecular weight | 992.09 |
| Space group (number) | $P2_1/n$ (14) |
| $\mu$ (mm$^{-1}$) | 6.473 |
| Temperature (K) | 170(100) |
| $Z$ | 4 |
| $\rho_{calc}$ (g/cm$^{-3}$) | 1.526 |
| Crystal size (mm$^3$) | 0.12×0.15×0.21 |
| Diffractometer | SuperNova, Dual, Cu at zero, Atlas |
| Radiation | Cu $K_\alpha$ |
| Total reflections | 71362 |
| Unique reflections | 8187 |
| Angle range 2$\Theta$ (°) | 6.50 to 140.00 (0.82 Å) |
| Reflections with $\|F_o\| \geq 4\sigma_F$ | 7906 |
| $R_{int}$ | 0.0406 |
| $R_\sigma$ | 0.0154 |
| $R_1$ ($\|F_o\| \geq 4\sigma_F$) | 0.0247 |
| $wR_2$ ($\|F_o\| \geq 4\sigma_F$) | 0.0645 |
| $R_1$ (all data) | 0.0254 |
| $wR_2$ (all data) | 0.0650 |
| Goodness-of-fit on $F^2$ | 1.107 |
| $\rho_{max}$, $\rho_{min}$ ($e$/Å$^3$) | 3.17/−0.62 |
| CCDC number | 2204302 |

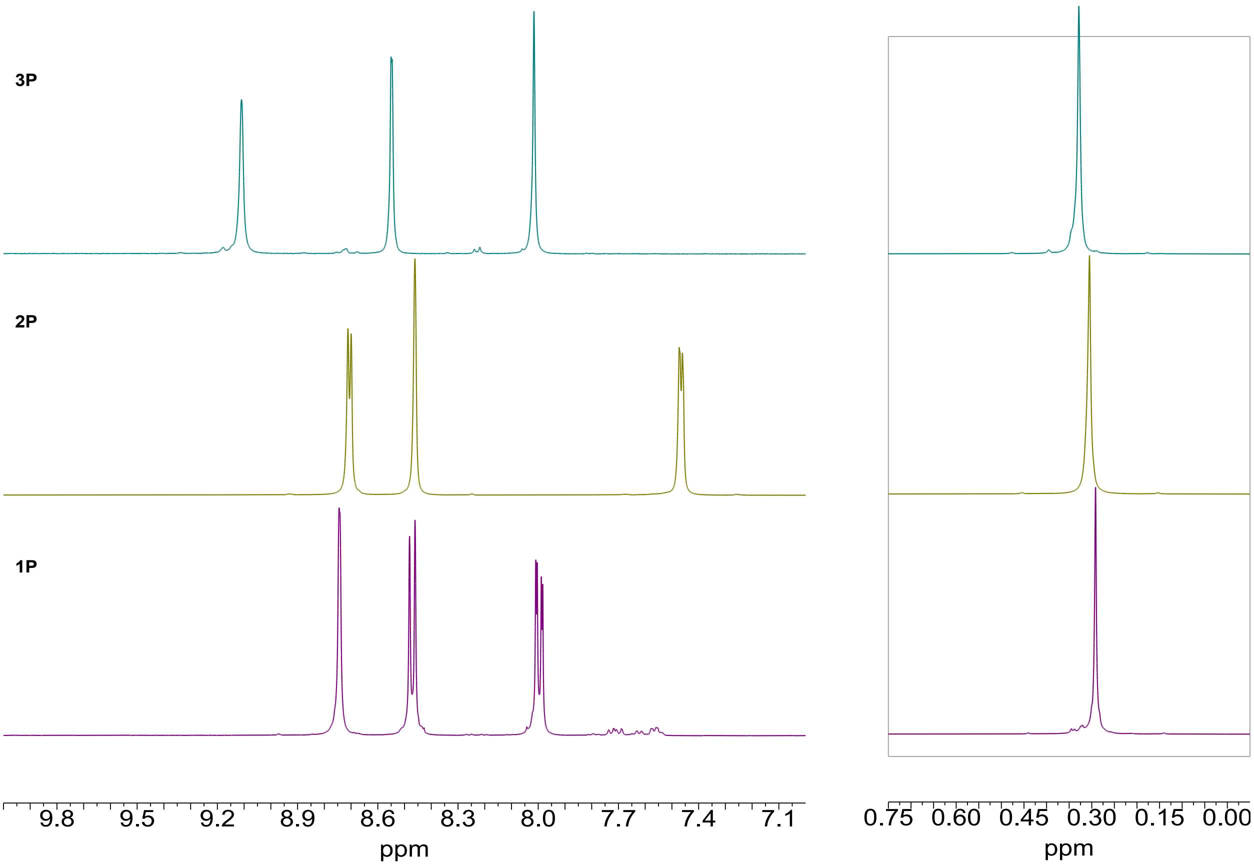


**Figure S1.** $^{1}$H NMR spectra of **NN** compounds **XP** (X = 1−3). Aromatic (left) and aliphatic (right) regions are shown on a different vertical scale.

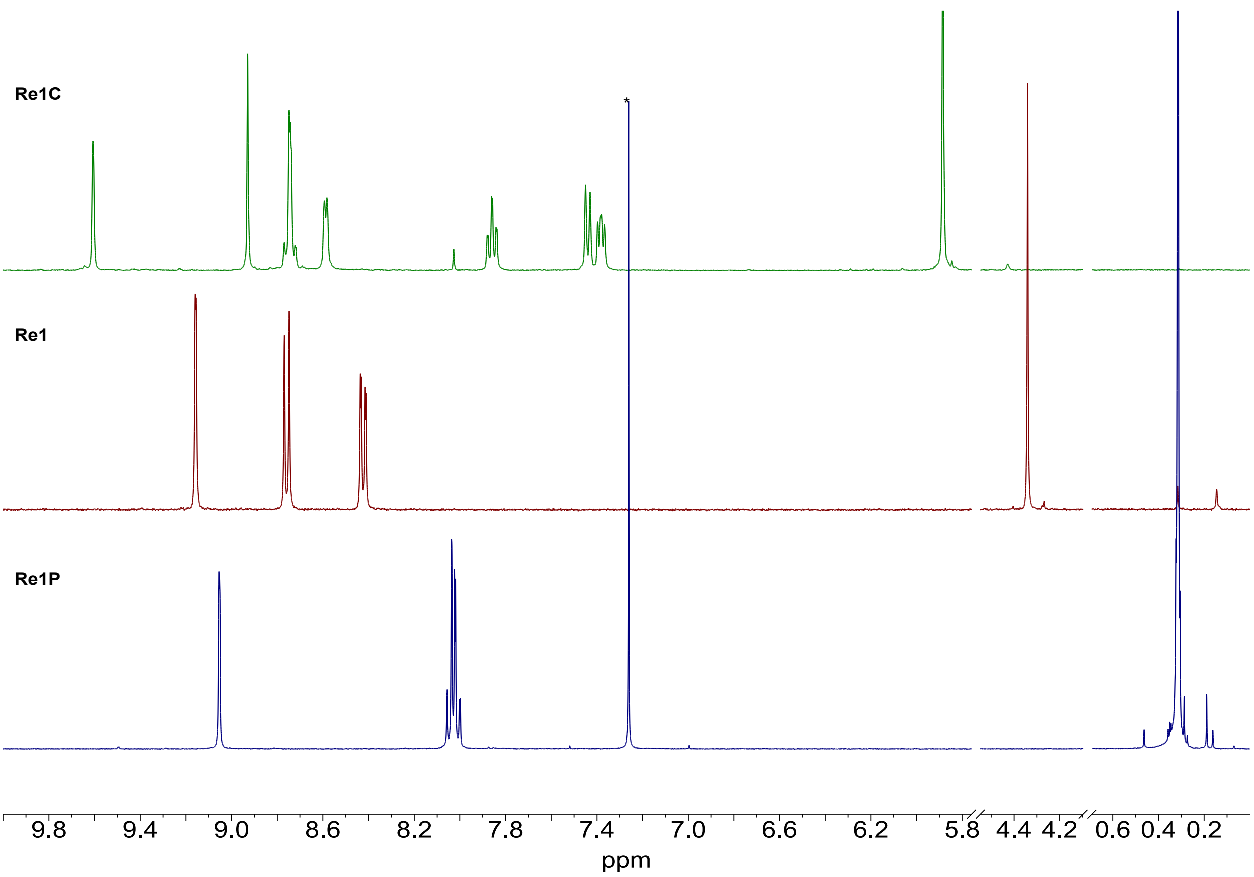


**Figure S2.** $^{1}H$ NMR spectra of **Re1$^{X}$** complexes. The residual solvent peak of $CDCl_3$ is marked by asterisk.

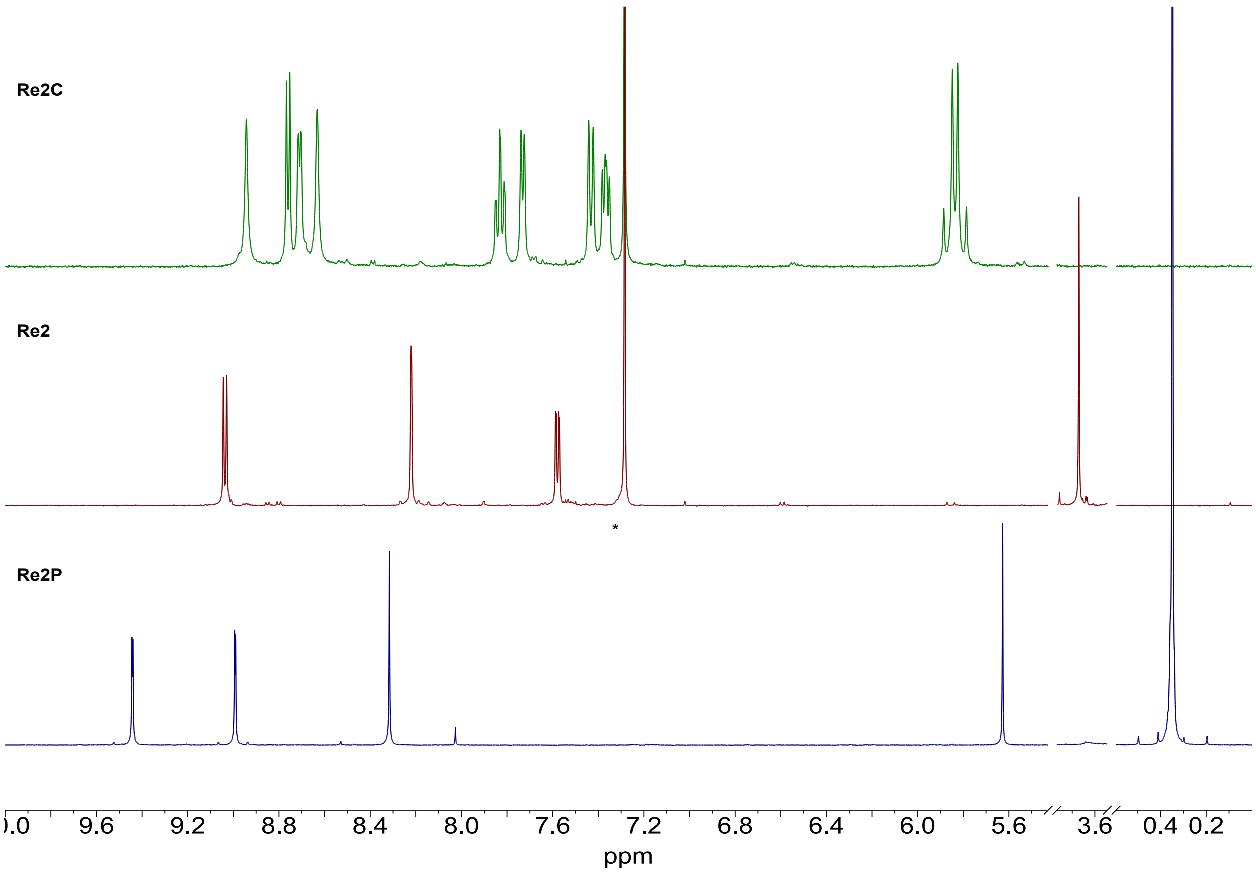


**Figure S3.** $^{1}$H NMR spectra of **Re2$^X$** complexes. The residual solvent peak of $CDCl_3$ is marked by asterisk.

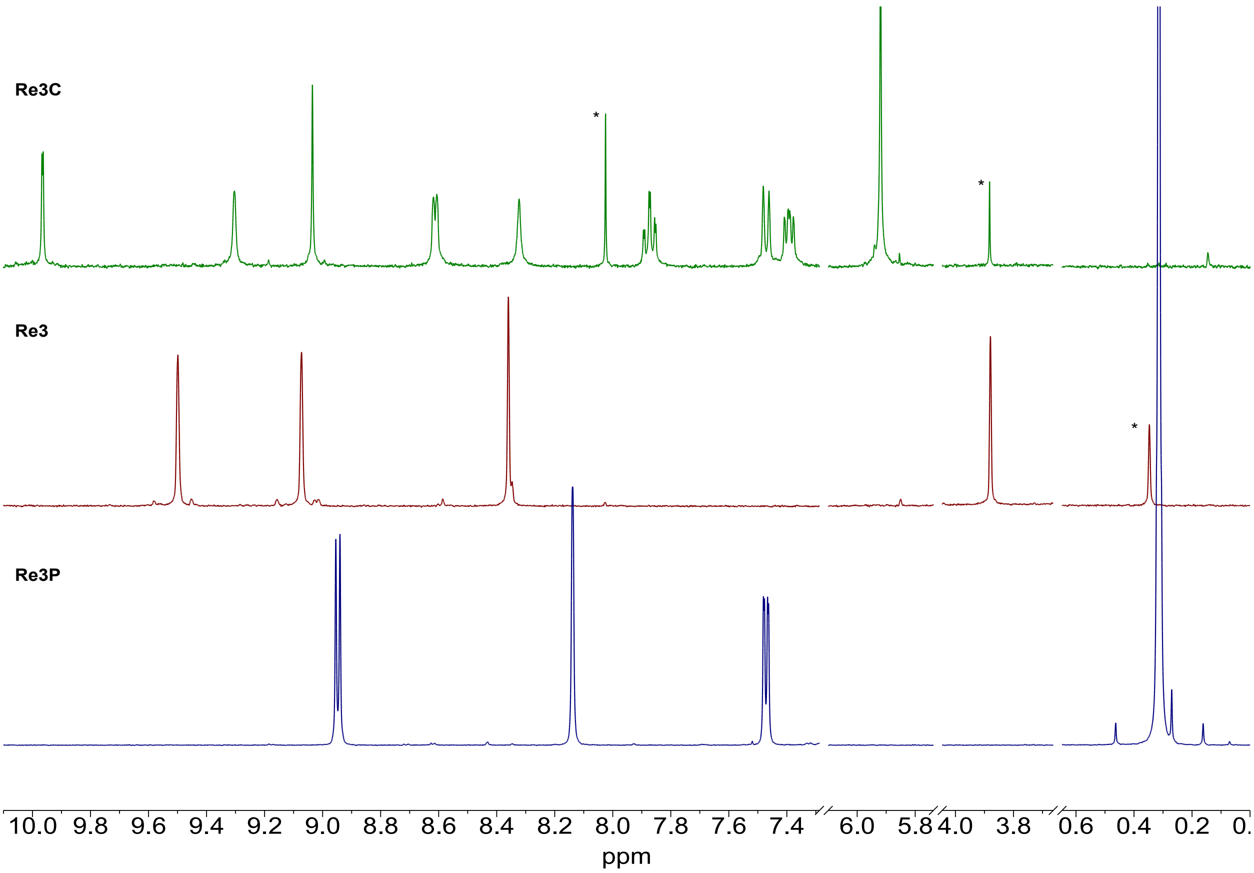


**Figure S4.** $^{1}H$ NMR spectra of **Re3$^{X}$** complexes. The resonances of admixtures are marked by asterisks.

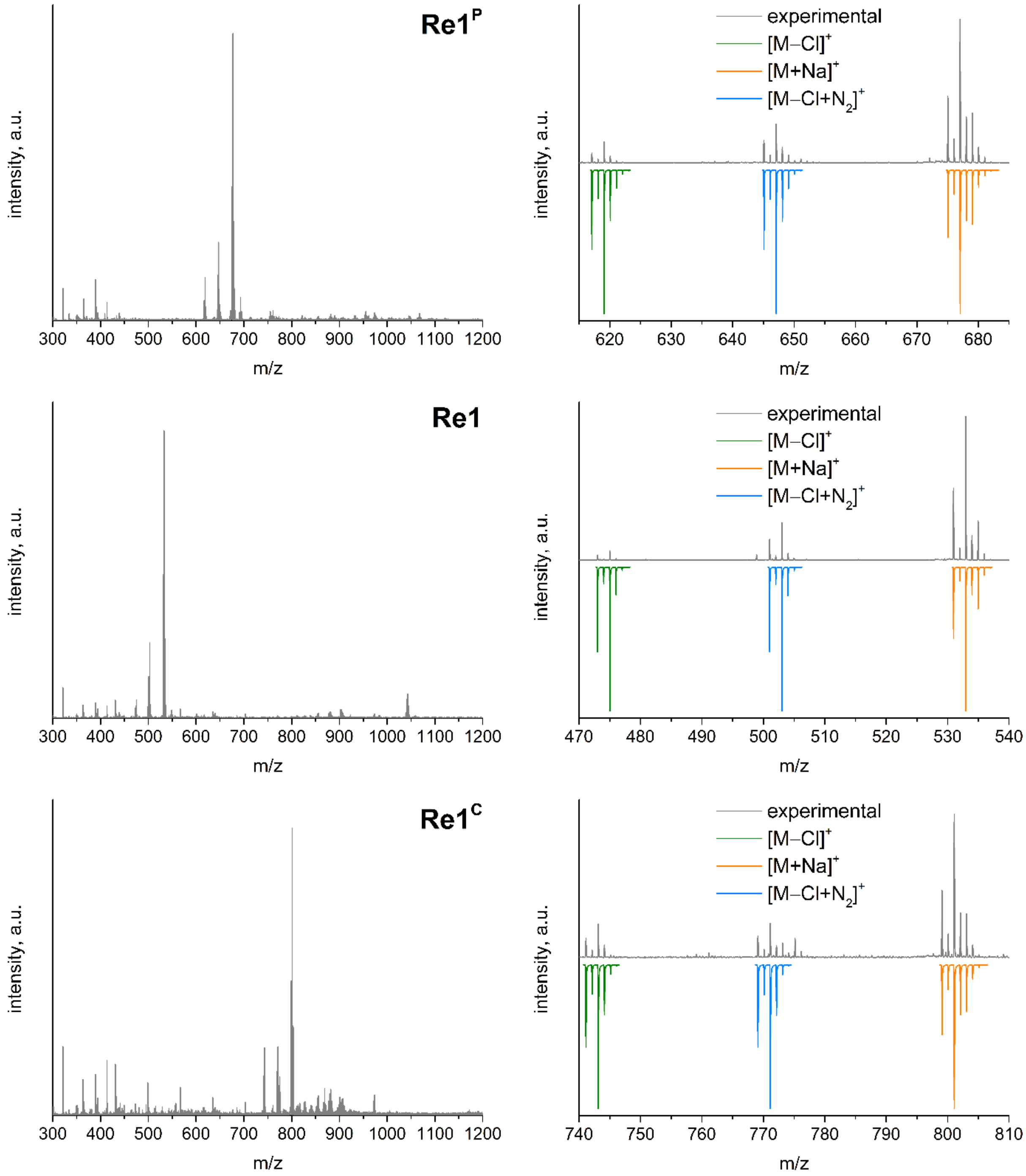


**Figure S5.** ESI$^{+}$ MS spectra of **Re1$^{X}$** complexes with isotope pattern of key signals.

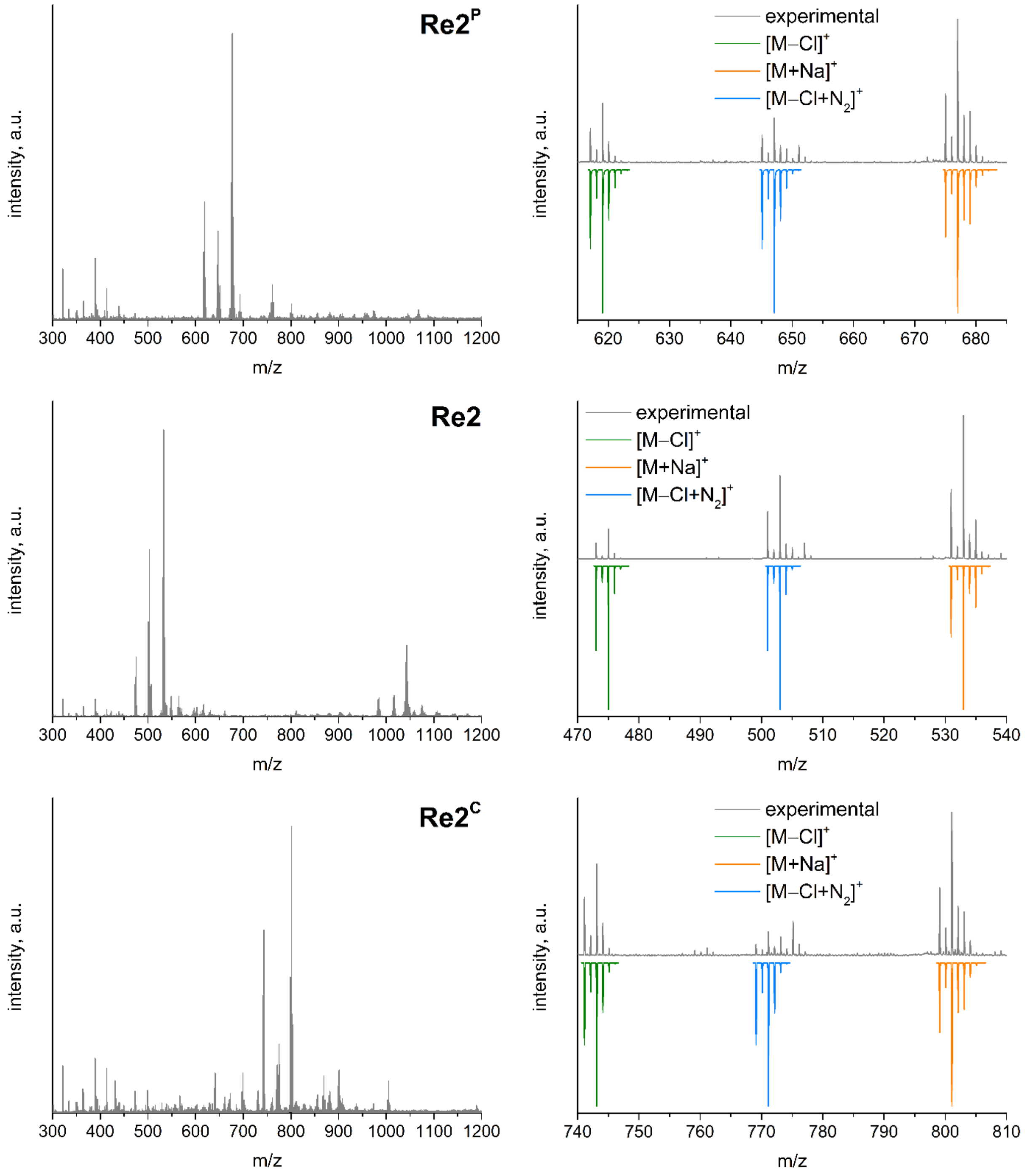


**Figure S6.** ESI$^+$ MS spectra of **Re2$^X$** complexes with isotope pattern of key signals.

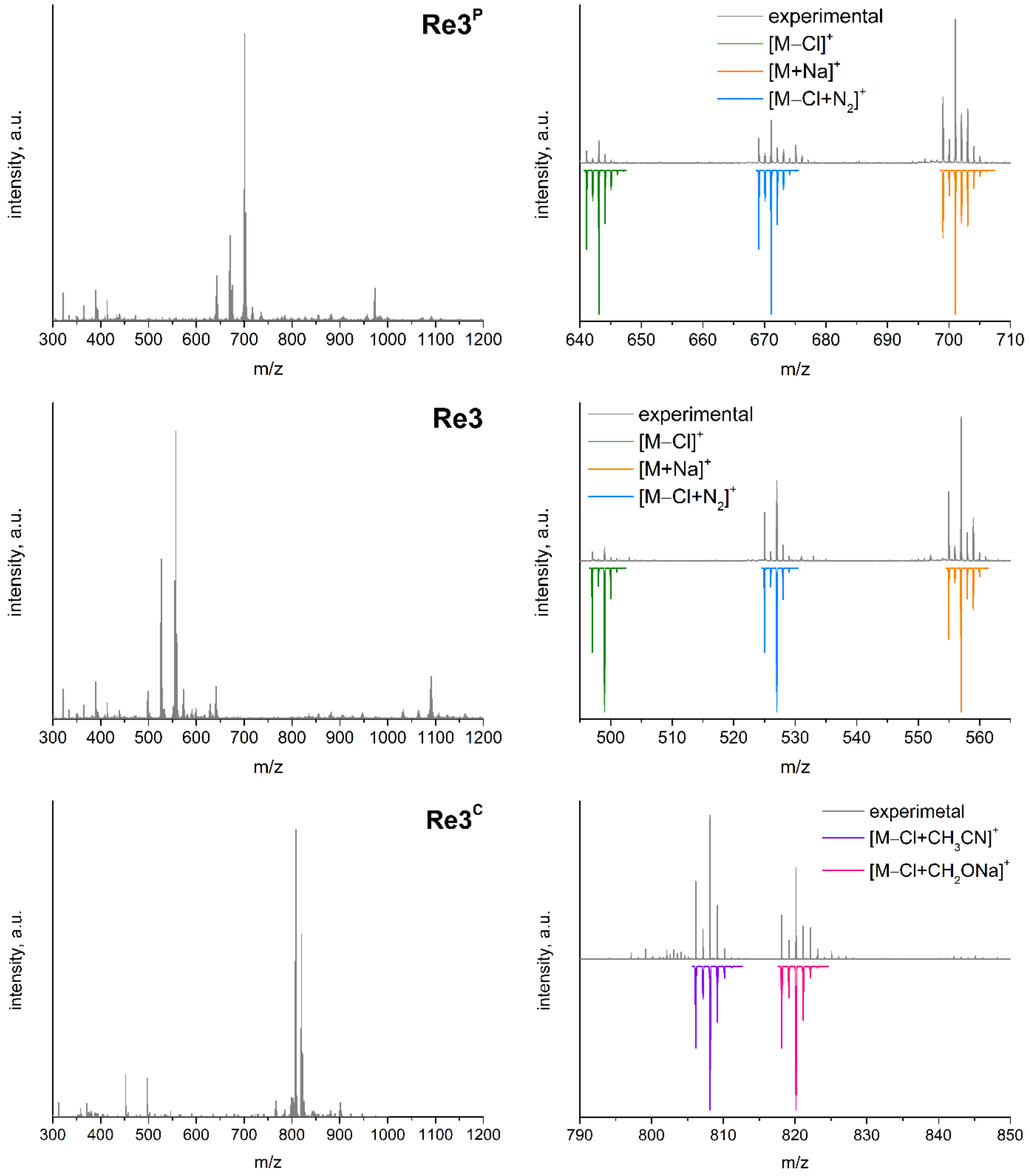


**Figure S7.** ESI$^+$ MS spectra of **Re3$^X$** complexes with isotope pattern of key signals.

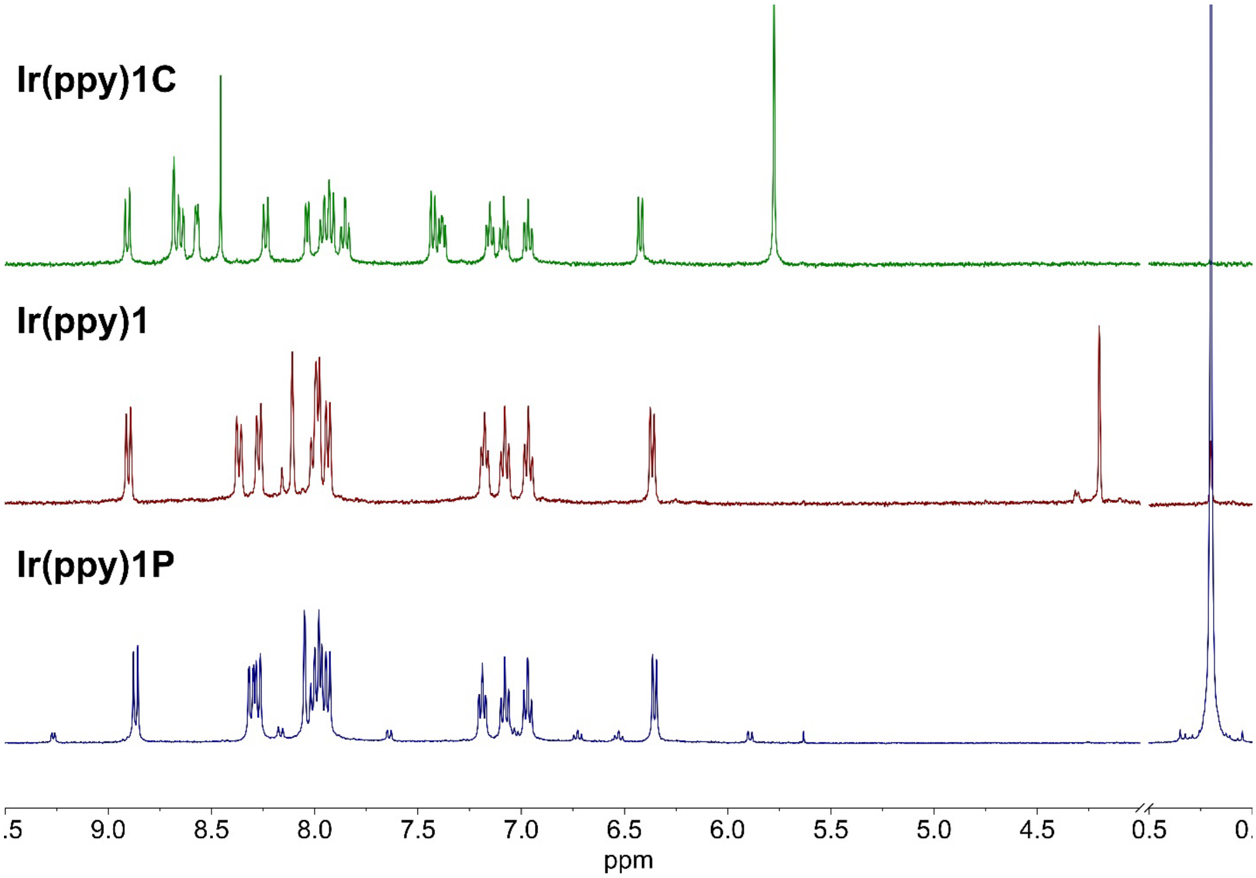


**Figure S8.** $^{1}$H NMR spectra of **Ir(ppy)1$^{X}$** complexes.

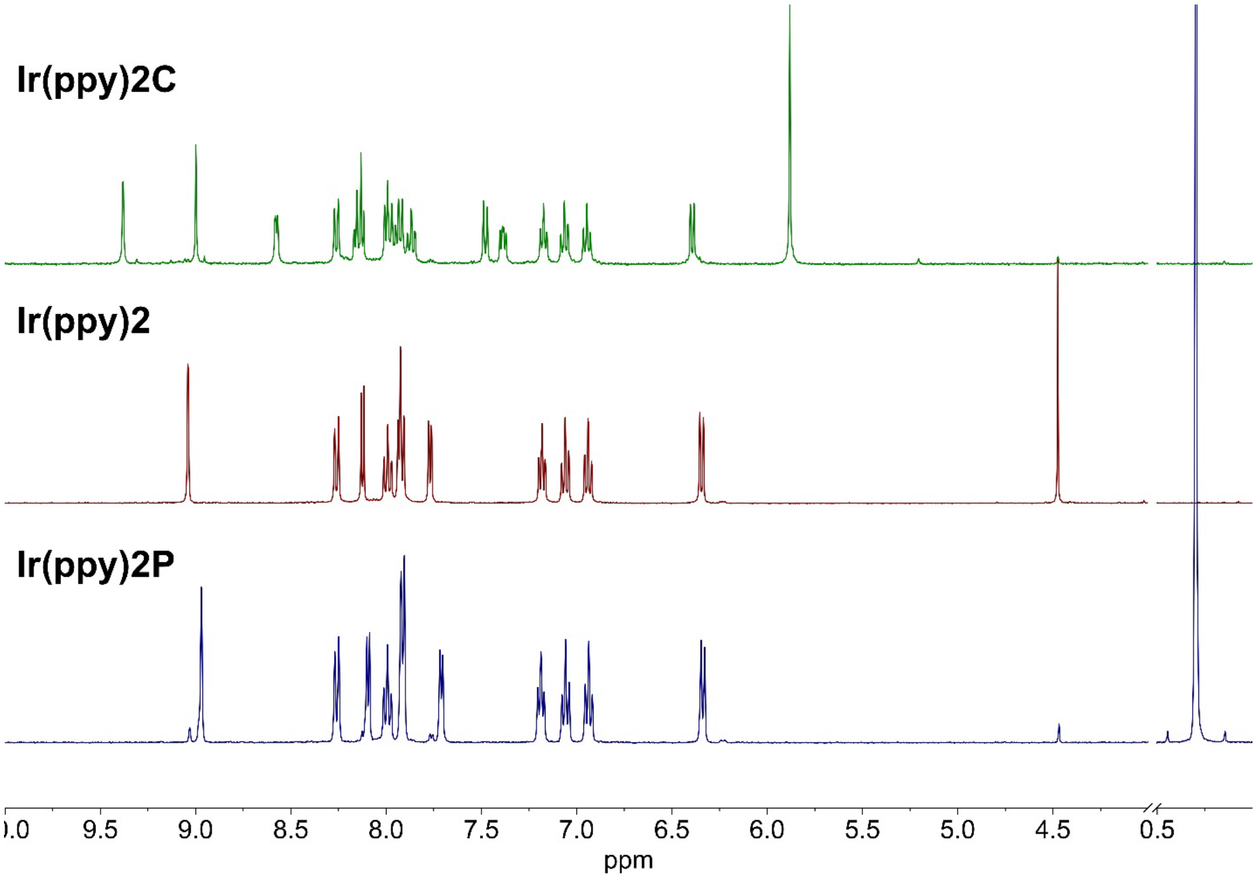


**Figure S9.** $^{1}$H NMR spectra of **Ir(ppy)2$^{X}$** complexes.

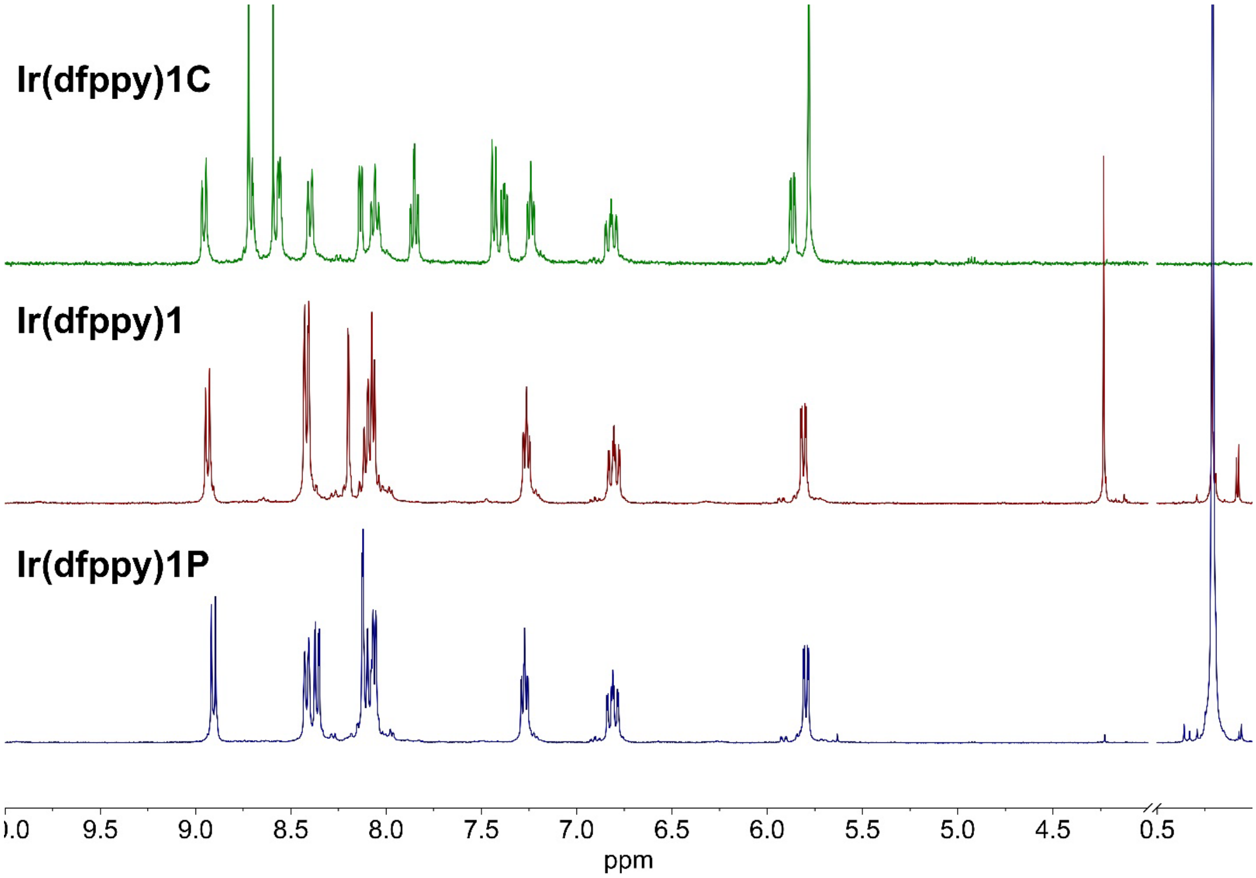


**Figure S10.** $^{1}$H NMR spectra of **Ir(dfppy)1$^{X}$** complexes.

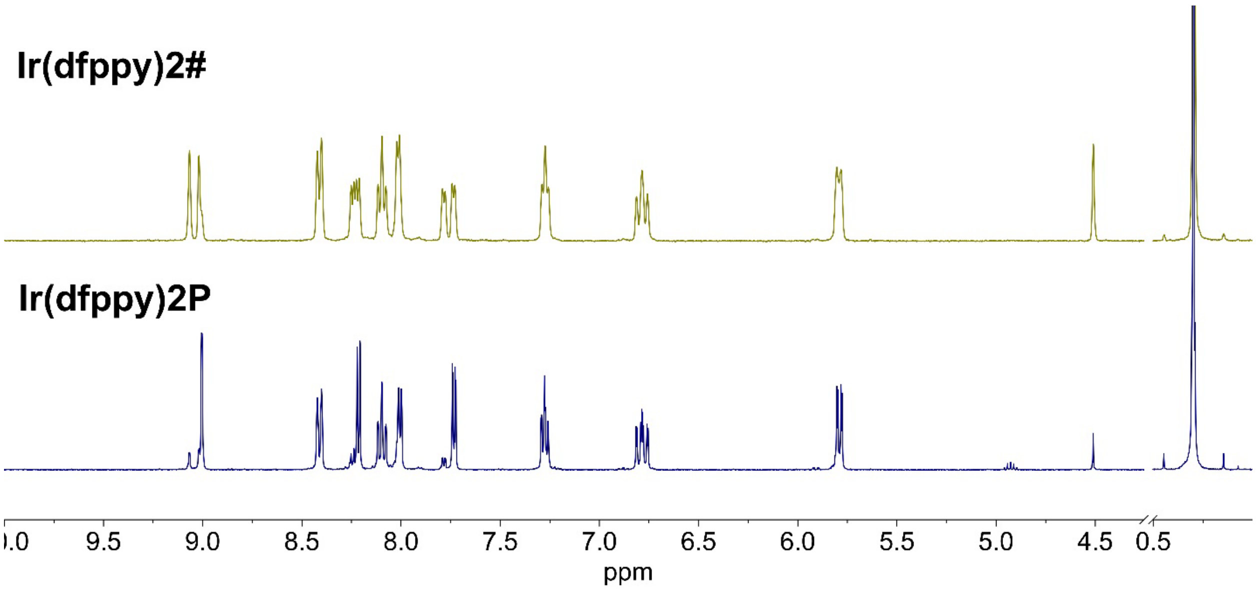


**Figure S11.** $^{1}$H NMR spectra of **Ir(dfppy)2$^{X}$** complexes.

#Mixture of **Ir(dfppy)2$^{P}$** and **Ir(dfppy)2**.

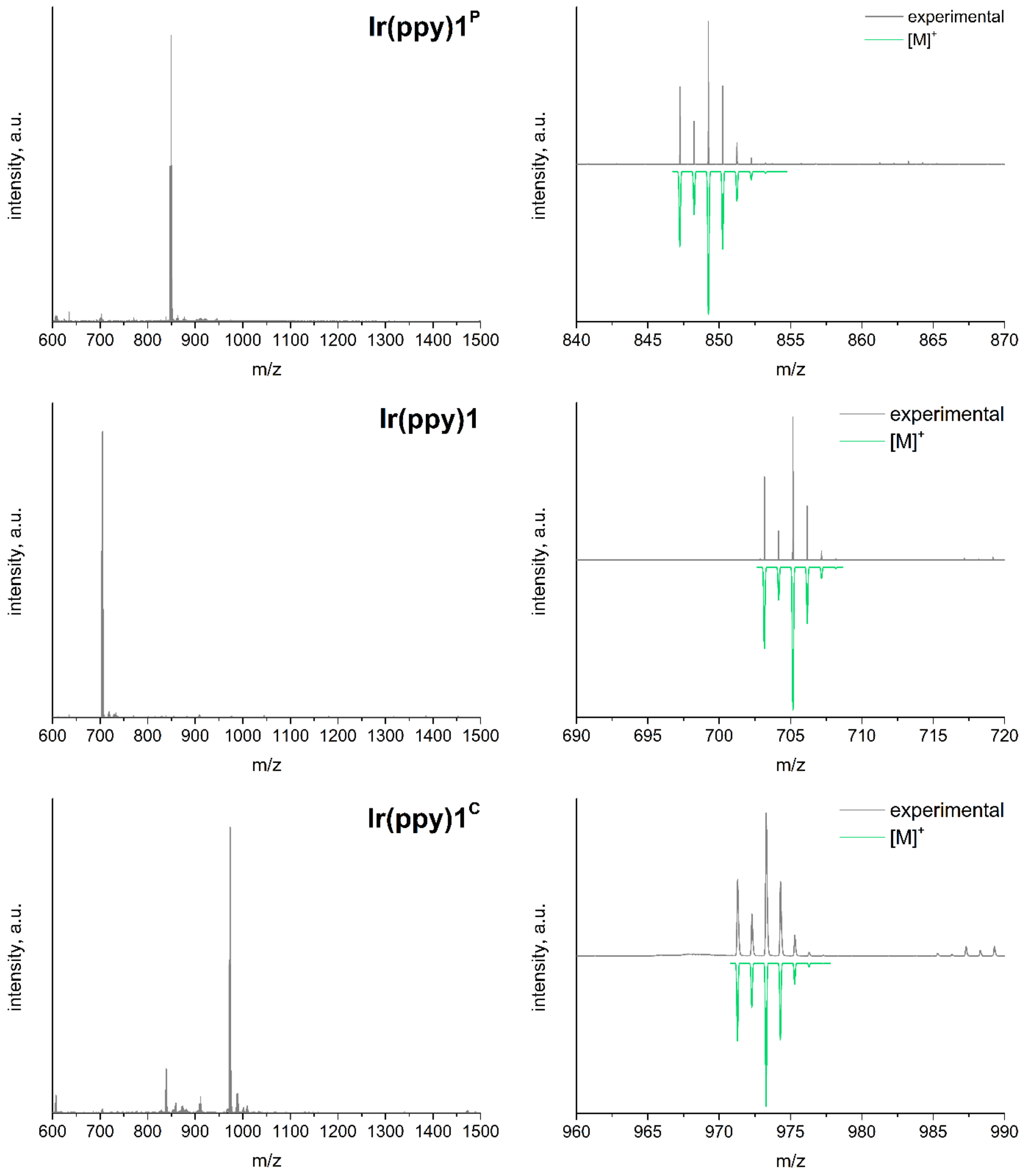


**Figure S12.** ESI$^+$ MS spectra of **Ir(ppy)1$^X$** complexes with isotope pattern of key signals.

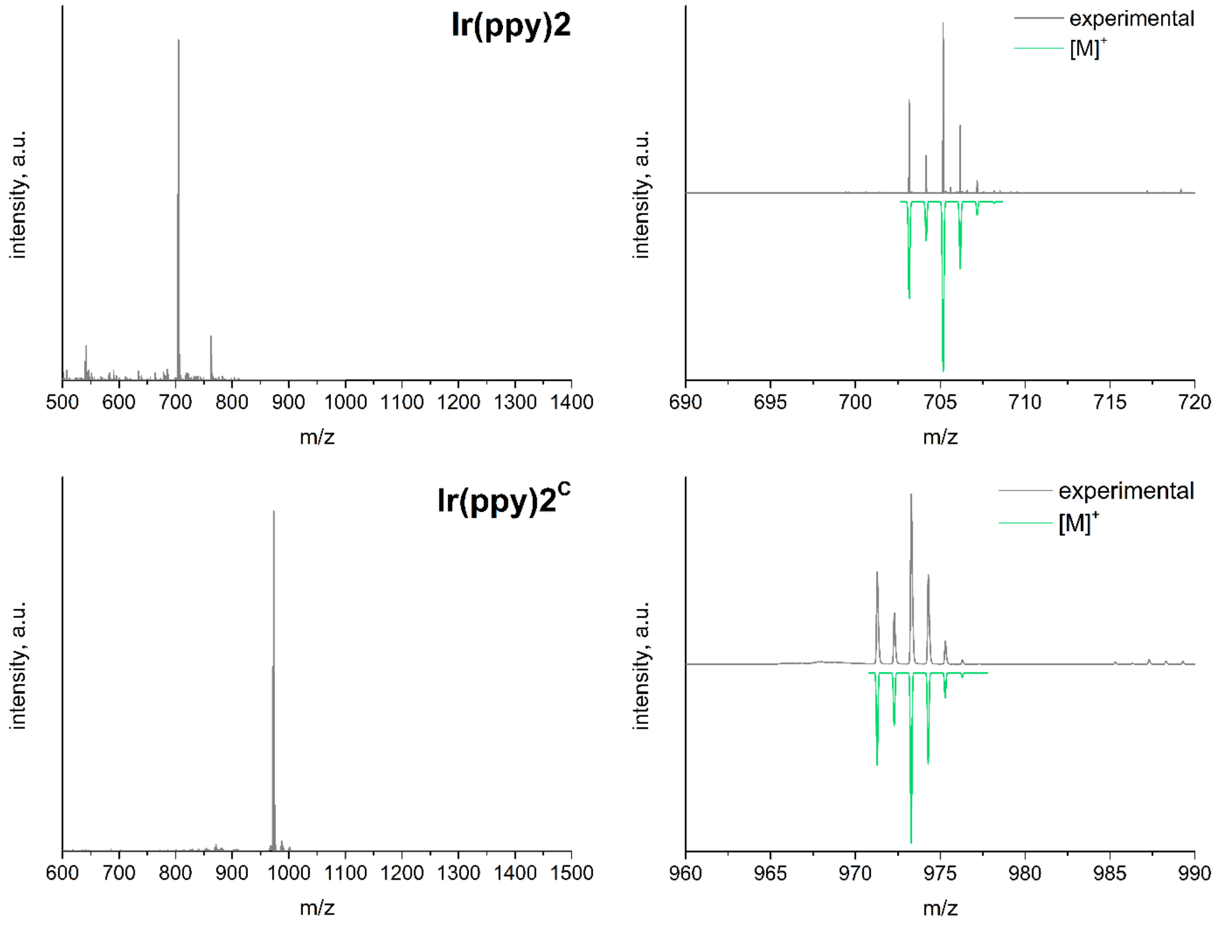


**Figure S13.** ESI$^+$ MS spectra of **Ir(ppy)2**$^{\mathbf{X}}$ complexes with isotope pattern of key signals.

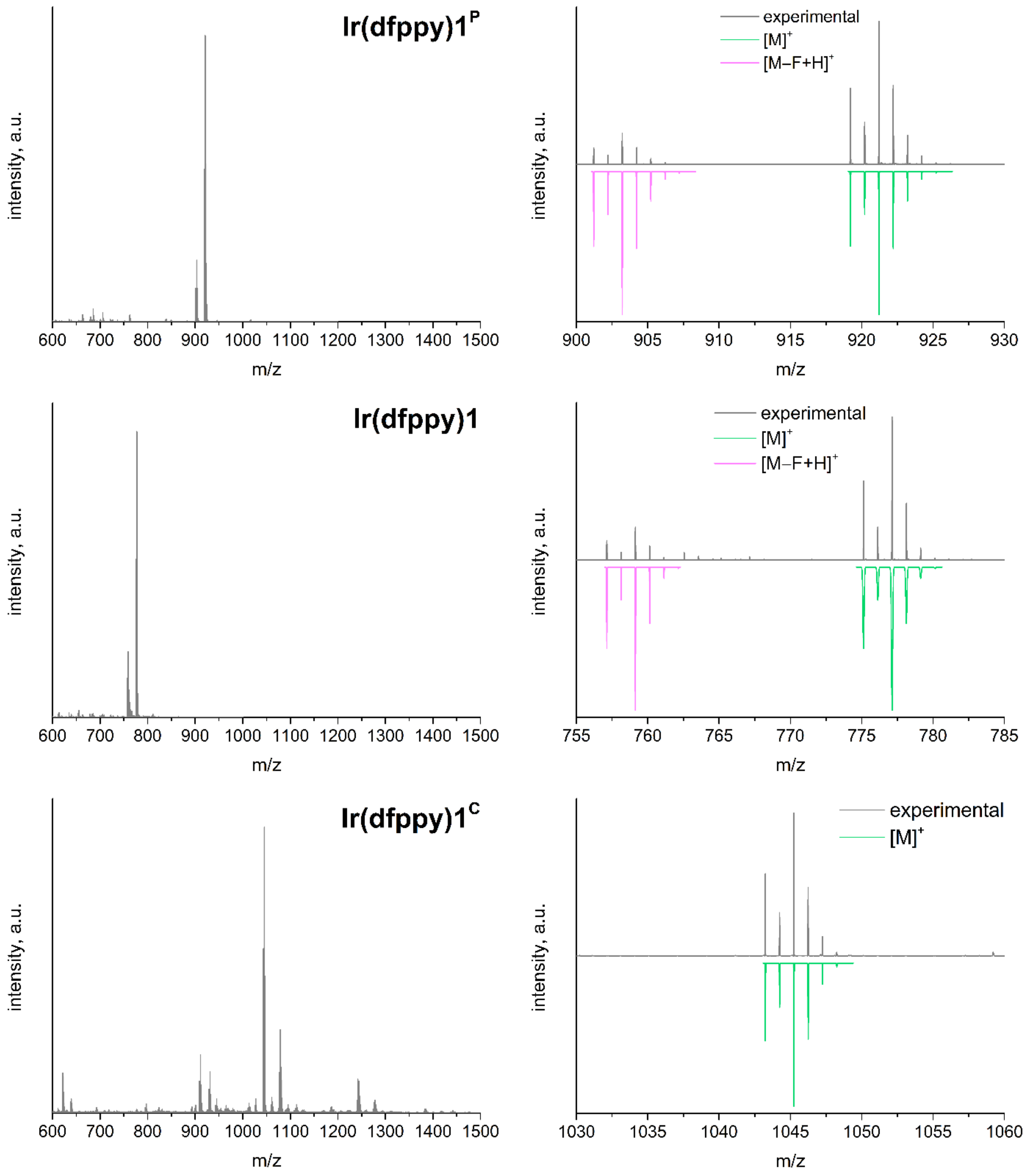


**Figure S14.** ESI$^+$ MS spectra of **Ir(dfppy)1**$^{\mathbf{X}}$ complexes with isotope pattern of key signals.

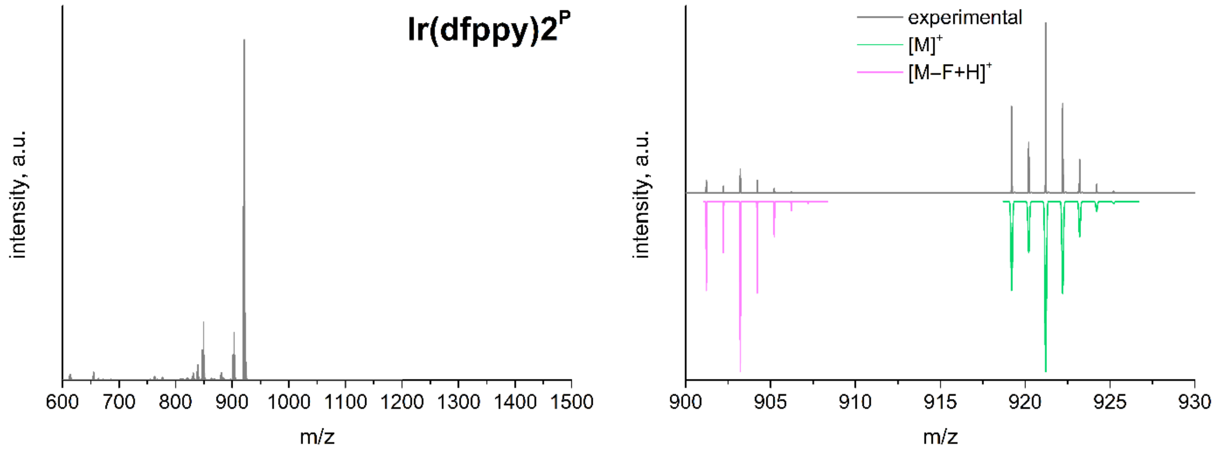


**Figure S15.** ESI$^+$ MS spectra of **Ir(dfppy)2$^P$** complexes with isotope pattern of key signals.

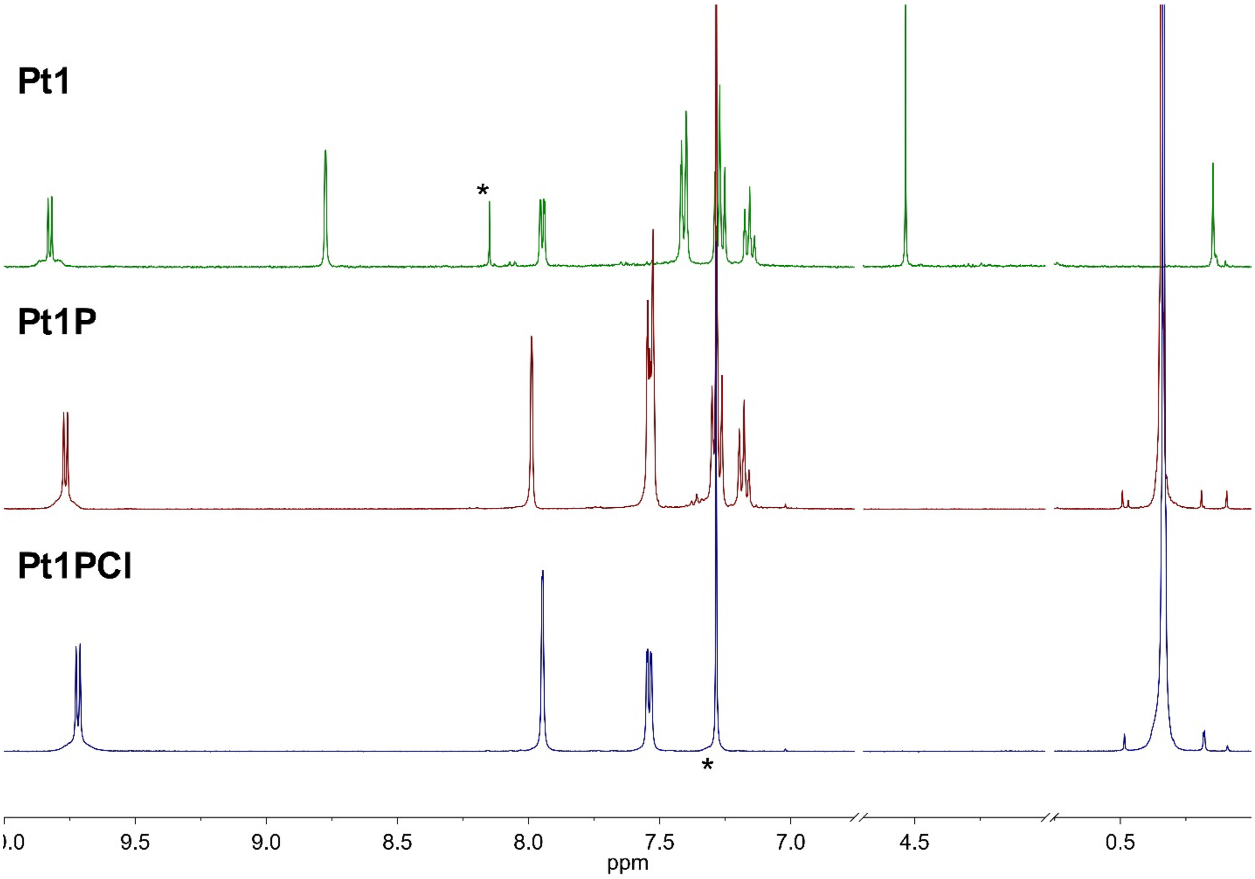


**Figure S16.** $^{1}$H NMR spectra of **Pt1^X^** complexes. Asterisks mark the resonances of admixtures and the residual solvent peak of $CDCl_3$ (7.29 ppm).

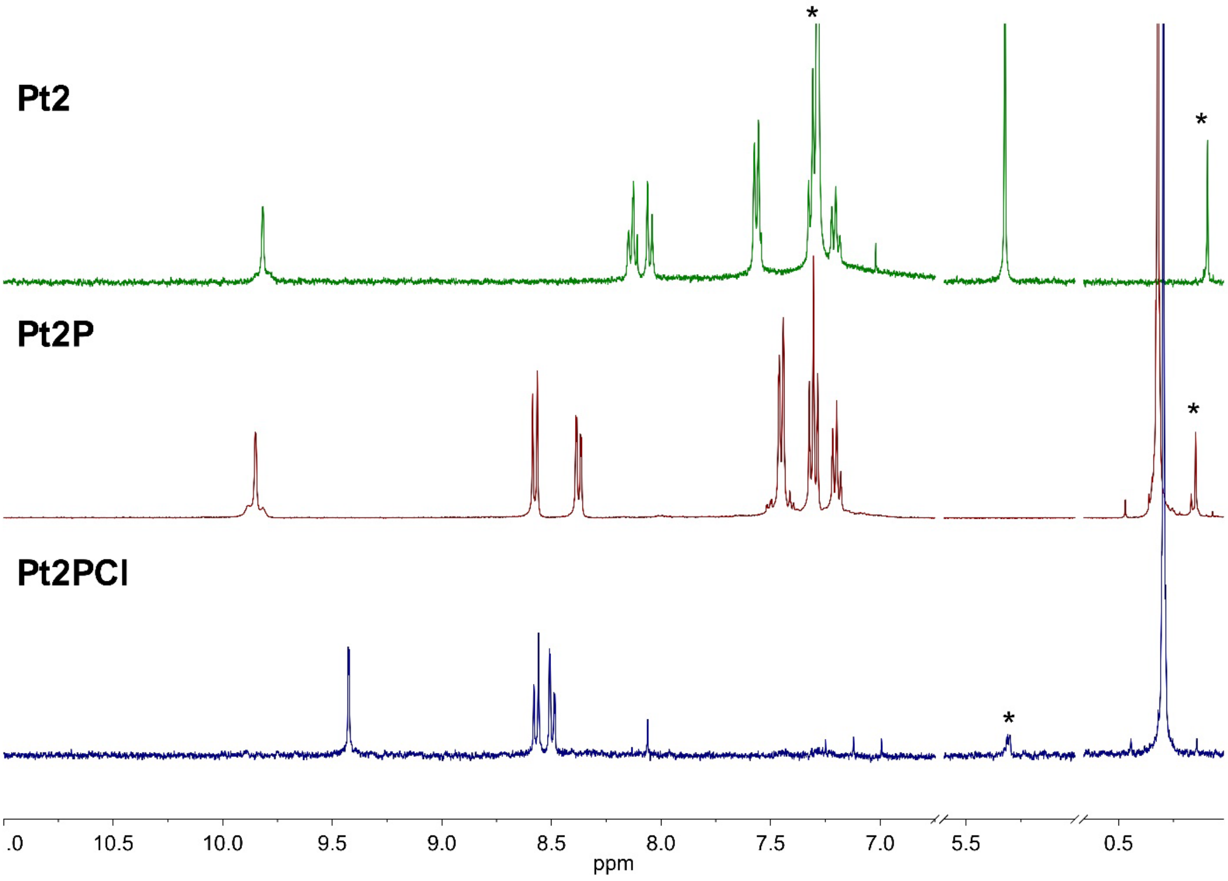


**Figure S17.** $^1$H NMR spectra of **Pt2**$^X$ complexes. Asterisks mark the resonances of admixtures and the residual solvent peak of $CDCl_3$ (7.29 ppm).

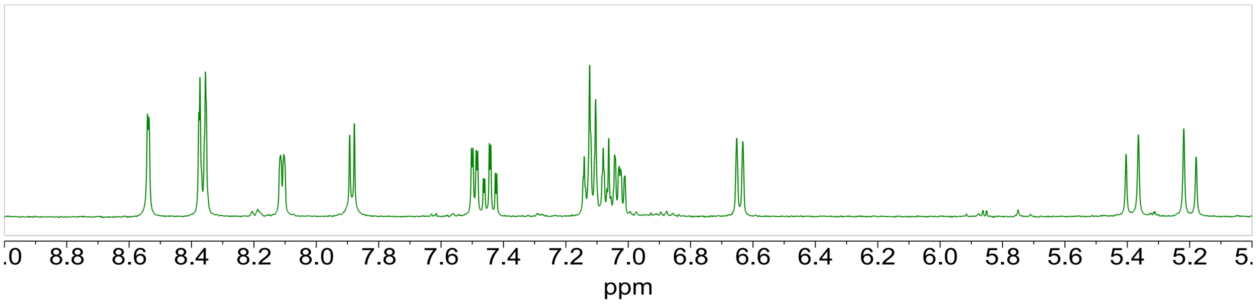


**Figure S18.** $^{1}$H NMR spectrum of **Pt(btbpy)$^{C}$** complexes in aromatic region, DMSO-$d_6$, RT.

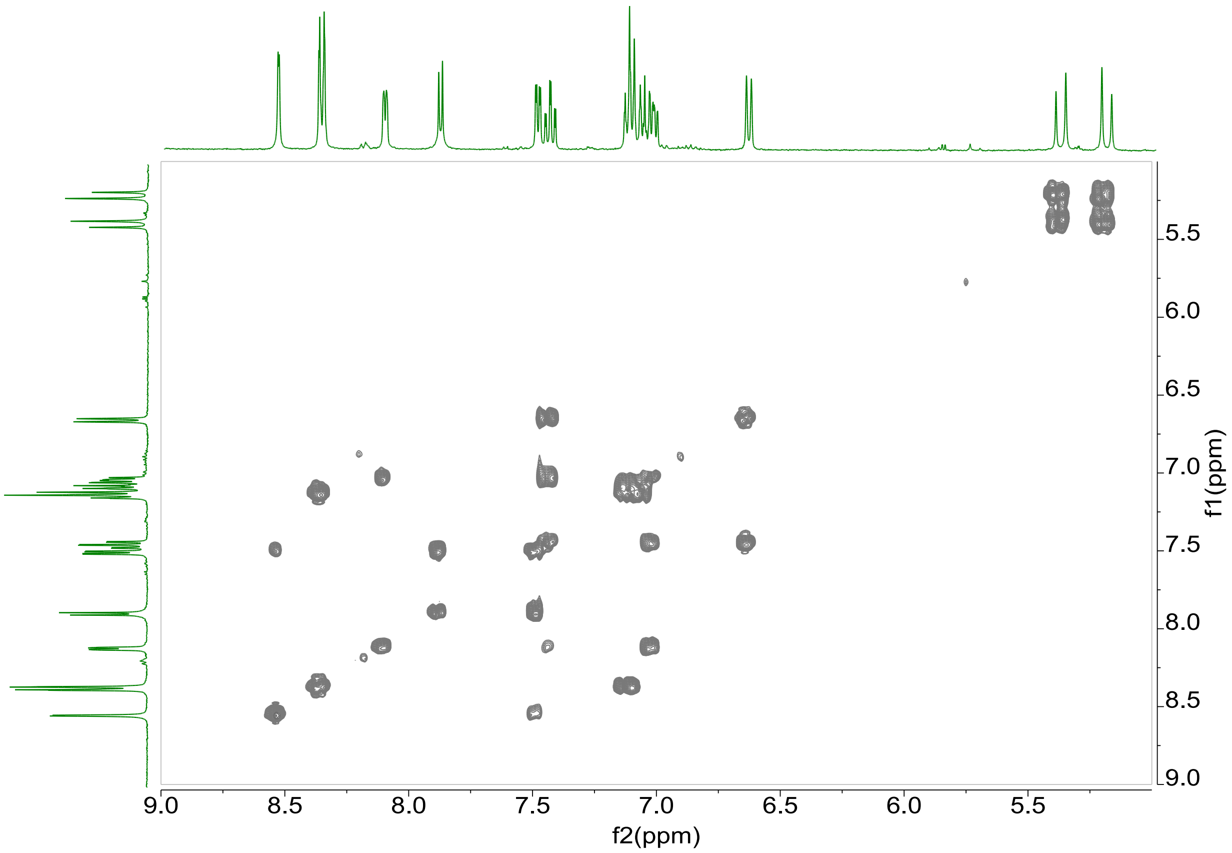


**Figure S19.** $^{1}$H$^{1}$H COSY NMR spectrum of **Pt(btbpy)$^{C}$** complexes in aromatic region, DMSO-$d_6$, RT.

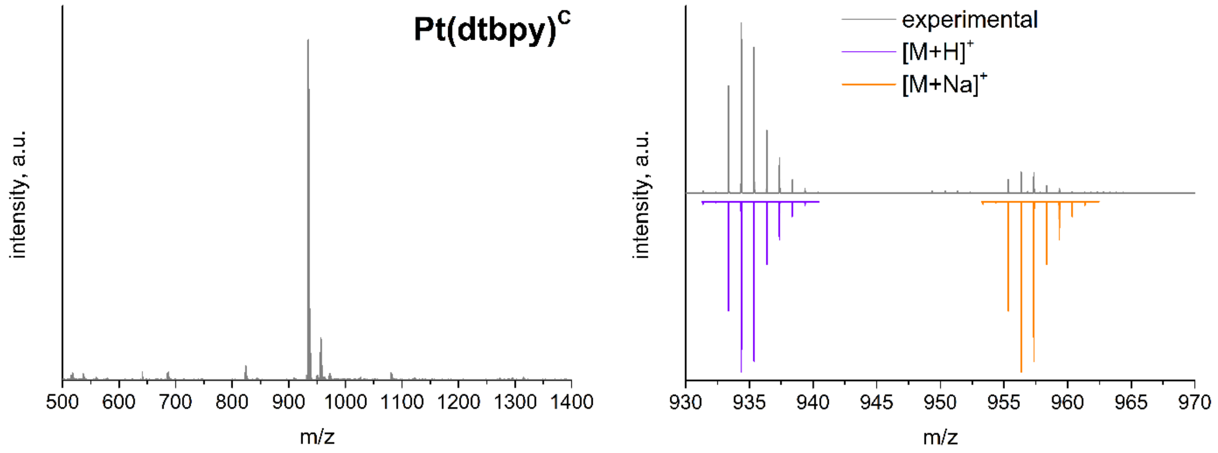


**Figure S20.** ESI$^+$ MS spectrum of **Pt(dtbpy)$^C$** complex with isotope pattern of key signals.

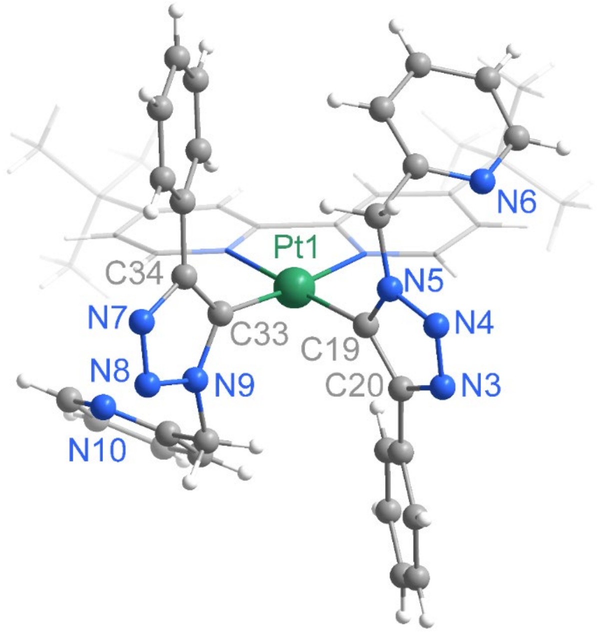


**Figure S21.** Molecular structure of **Pt(dtbpy)$^C$** complex with key atoms numeration.

**Table S2.** Selected structural parameters for compound **Pt(dtbpy)$^{C}$** and literature data for Pt(II) triazolyl derivatives [5,6].

| Bond lengths, Å | | Literature reported range, Å |
|---|---|---|
| Pt1–N1 | 2.078(2) | 1.959 – 2.077 |
| Pt1–N2 | 2.079(2) | |
| Pt1–C19 | 2.010(2) | 1.989 – 2.007 |
| Pt1–C33 | 2.001(3) | |
| C19–C20 | 1.394(4) | 1.376 – 1.407 |
| C33–C34 | 1.394(4) | |
| N3–N4 | 1.305(3) | 1.306 – 1.322 |
| N7–N8 | 1.309(3) | |
| N4–N5 | 1.354(3) | 1.351 – 1.386 |
| N8–N9 | 1.354(3) | |
| **Bond angles, °** | | **Literature data, °** |
| N1–Pt1–N2 | 78.29 (9) | 76.86 |
| C19–Pt1–C40 | 88.02(10) | 88.55 |
| **Plane angle, °** | | **Literature data, °** |
| Pt-N1-N2 ring–C19 ring | 95.543 | 87.38 |
| Pt-N1-N2 ring–C33 ring | 95.586 | |

**Table S3.** Photophysical properties of "clicked" complexes **ReX$^{C}$** and **IrX$^{C}$**, DCE, r.t., $10^{-5}$ M.

| | **λ, nm (ε, $10^{-3}$ cm$^{-1}$M$^{-1}$)** | **$λ_{em}$, nm** | **Φ, %** | | **τ, μs** | |
|---|---|---|---|---|---|---|
| | | | A | D | A | D |
| **Re1$^{C}$** | 291 (28.5), 351 (31.8), 365 (32) | 660 | <1 | <1 | 0.24 | 0.22 |
| **Re2$^{C}$** | 291 (43.4) | 650 | <1 | <1 | 0.49 | 0.52 |
| **Re3$^{C}$** | 280 (47.8), 342 (31.8) | 645 | <1 | <1 | 0.88 | 0.87 |
| **Ir(dfppy)1$^{C}$** | 252 (33.9), 307 (25.3), 357 (26.1) | 533 | 7.7 | 42.7 | 0.79 | 2.14 |
| **Ir(ppy)1$^{C}$** | 254 (65.7), 297 (50.8), 343 (42.5), 357 (43.1) | 626 | 7.7 | 19.2 | 0.22 | 0.35 |
| **Ir(ppy)2$^{C}$** | 267 (94.4), 365 (18.8) | 609 | 7.1 | 37.9 | 0.22 | 0.42 |

A = aerated. D = degassed.

**References**


1. *CrysAlisPro, Rigaku Oxford Diffraction*; Agilent Technologies: Version 1.171.39.35a, 2017;
2. Sheldrick, G.M. SHELXT - Integrated space-group and crystal-structure determination. *Acta Crystallogr. Sect. A Found. Crystallogr.* **2015**, *71*, 3–8, doi:10.1107/S2053273314026370.
3. Sheldrick, G.M. Crystal structure refinement with SHELXL. *Acta Crystallogr. Sect. C* **2015**, *71*, 3–8, doi:10.1107/S2053229614024218.
4. Dolomanov, O. V.; Bourhis, L.J.; Gildea, R.J.; Howard, J.A.K.; Puschmann, H. OLEX2 : a complete structure solution, refinement and analysis program. *J. Appl. Crystallogr.* **2009**, *42*, 339–341, doi:10.1107/S0021889808042726.
5. Li, Y.; Tsang, D.P.; Chan, C.K.; Wong, K.M.; Chan, M.; Yam, V.W. Synthesis of Unsymmetric Bipyridine–Pt II –Alkynyl Complexes through Post-Click Reaction with Emission Enhancement Characteristics and Their Applications as Phosphorescent Organic Light-Emitting Diodes. *Chem. – A Eur. J.* **2014**, *20*, 13710–13715, doi:10.1002/chem.201404315.
6. Guha, R.; Defayay, D.; Hepp, A.; Müller, J. Targeting Guanine Quadruplexes with Luminescent Platinum(II) Complexes Bearing a Pendant Nucleobase. *Chempluschem* **2021**, *86*, 662–673, doi:10.1002/cplu.202100135.
